# Thermoelectric–Photoelectrochemical Water Splitting under Concentrated Solar Irradiation

**DOI:** 10.1021/jacs.3c01892

**Published:** 2023-06-13

**Authors:** Chanon Pornrungroj, Virgil Andrei, Erwin Reisner

**Affiliations:** † Yusuf Hamied Department of Chemistry, University of Cambridge, Lensfield Road, Cambridge CB2 1EW, United Kingdom

## Abstract

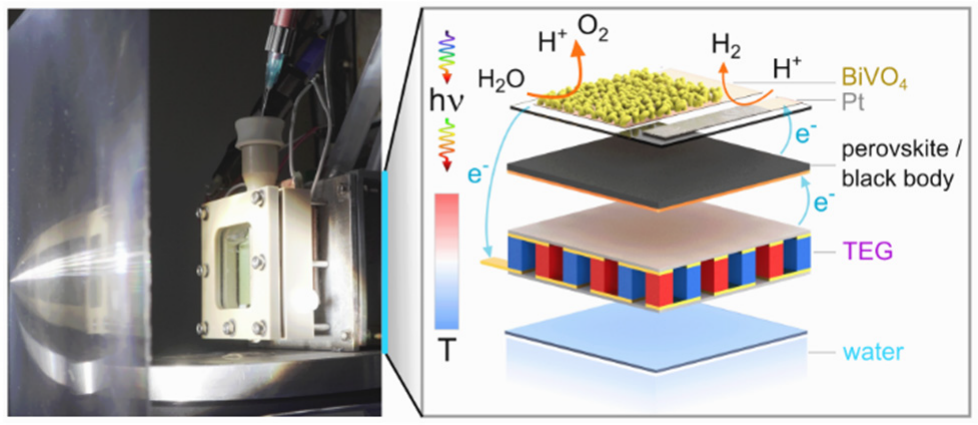

Photoelectrochemical devices could play a crucial role toward fuel production in a circular economy. Yet, light absorption suffers losses from thermalization and the inability to use low-energy photons. Here, we demonstrate that photoelectrochemical reactors can utilize this waste heat by integrating thermoelectric modules, which provide additional voltage under concentrated light irradiation. While most single semiconductors require external bias, we already accomplish unassisted water splitting under 2 sun irradiation by wiring a BiVO_4_ photoanode to a thermoelectric element, whereas the photocurrent of a perovskite-BiVO_4_ tandem system is enhanced 1.7-fold at 5 sun. This strategy is particularly suitable for photoanodes with more positive onset potentials like hematite, with thermoelectric-perovskite-Fe_2_O_3_ systems achieving a 29.7× overall photocurrent increase at 5 sun over conventional perovskite-Fe_2_O_3_ devices without light concentration. This thermal management approach provides a universal strategy to facilitate widespread solar fuel production, as light concentration increases output, reduces the reactor size and cost, and may enhance catalysis.

## Introduction

A broad utilization of the solar spectrum is key for efficient solar fuel production.^1,2^ Yet, light absorbers are limited by the maximum achievable efficiency known as the Shockley–Queisser limit.^3^ Hence, over 50% of the total energy is lost through thermalization or from nonabsorbed visible and IR photons with energies below the semiconductor bandgap.^1,3^ The resulting heating is known to decrease the photovoltage and promote photovoltaic (PV) cell degradation.^4^ However, higher temperatures can also prove beneficial by accelerating the reaction rate of electrocatalysis in solar fuel production.^4^ Strategies such as up- and downconversion have been employed to overcome this limitation, but challenges remain in the overall system integration and material stability under ambient conditions.^5−7^

A complementary route to harvest this waste heat is presented by thermoelectrics (TEs).^2^ In this case, a potential difference is induced along a material in the presence of a temperature gradient, which is known as the Seebeck effect.^8−10^ An array of such p- and n-type doped semiconductors can be assembled electrically in series and thermally in parallel to form a thermoelectric generator (TEG, see scheme in Figure S1).^8^ Its additional Seebeck voltage can be particularly useful in aiding photoelectrochemical (PEC) fuel production, as the thermodynamic requirements, catalyst overpotentials, and band positions of conventional photoelectrodes make overall water splitting challenging.

While a number of solar concentrator TE^11,12^ and TE-PV^13,14^ systems prove the utility of this approach for electricity production, few reports proposed TE systems for solar fuel synthesis.^2,15,16^ However, earlier TE-PEC studies only investigated photoelectrodes under 1 sun irradiation, meaning that the temperature difference (Δ*T*) was controlled by active heating requiring an external energy input. Accordingly, a decoupled TE-Si-Pt design needed an externally set Δ*T* above 60 K to overcome >2 V for overall water splitting,^16^ whereas a more integrated TE-Si-BiVO_4_ system only attained a light-induced Δ*T* of 9 K due to its side-on irradiation.^15^

**Figure 1:**
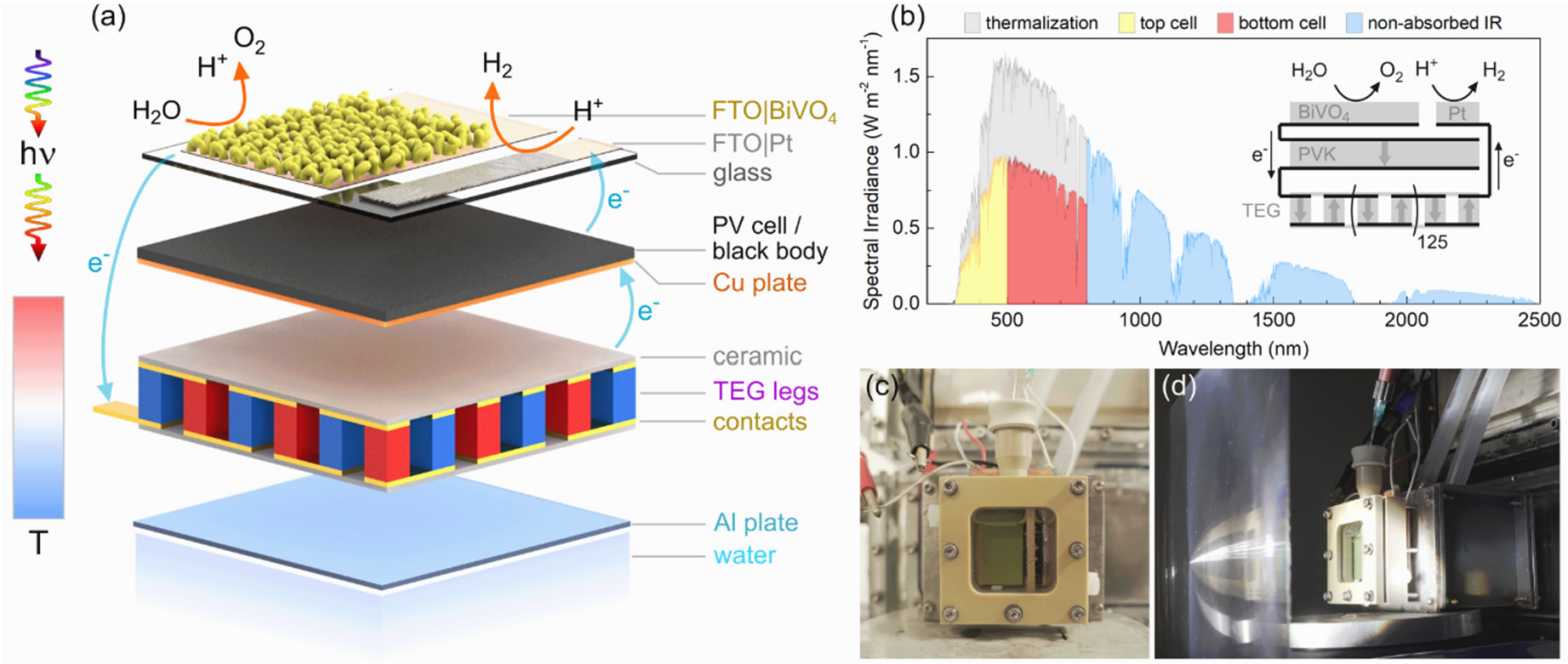
Architecture of the integrated thermoelectric–photoelectrochemical setup for solar water splitting. (a) Expanded schematic of the device assembly. The BiVO_4_ photoanode and Pt cathode are deposited on patterned FTO glass. Higher wavelength light is transmitted to either a perovskite PV cell or a blackbody converting light into heat. This temperature difference between the light absorbers and water bath is harvested by a TEG to provide additional voltage. (b) Solar spectrum (ASTM G173-03 AM1.5G reference spectrum) utilization by a tandem light harvesting device. Incident energy is lost as waste heat from thermalization or nonabsorbed photons (simplified schematic is plotted assuming 60% EQE for both light absorbers). The inset depicts the wiring of an integrated TE-PEC device. The BiVO_4_ photoanode, perovskite cell, and Pt cathode are wired electrically in series to a commercial TEG, which is comprised of 127 TE leg pairs. (c, d) Photographs of the assembled reactor without irradiation (c) and operating under concentrated light irradiation (d). A separator is mounted between the BiVO_4_ and Pt electrodes (c). The Fresnel lens and rear water bath help sustain the temperature difference (d).

Here, we introduce an integrated TE-PEC design for overall water splitting under concentrated solar irradiation. This design makes use of the entire spectral range, by placing the single or tandem light absorbers and heat-harvesting TEG in a single optical light path (Figure 1a,b). In this case, a steady temperature gradient is sustained via passive heating, by conveniently placing the commercial TEG between a hot PEC reactor and a room-temperature water bath emulating the open water sources (e.g., lakes, rivers) used in industrial cooling (Figure 1). This setup benefits from the higher temperatures attained under light concentration, as unassisted water splitting is already achieved when interfacing a commercial TEG with a single light absorber, a BiVO_4_ photoanode. A broadband coverage of the solar spectrum, from UV to the IR range, is demonstrated when introducing an additional perovskite (PVK) PV cell between the BiVO_4_ and TE components. The applicability of this thermal management strategy to various light absorbers is exemplified using hematite (α-Fe_2_O_3_), as TE-PVK-Fe_2_O_3_ assemblies display a significant photocurrent increase under light concentration despite the lower hematite photovoltage. This truly integrated circuit makes use of charge, photon, and heat flows, demonstrating the potential of overall energy management.

## Results and Discussion

### TEG-Photoanode Integration

The energy levels required for overall water splitting limit the choice of materials to a small selection of wide bandgap semiconductors. To demonstrate the benefits of our heat harvesting concept, we first assembled a TE-PEC system containing a single light absorber. For this purpose, we chose a robust BiVO_4_ photoanode, which displays high photocurrents reaching 3 mA cm^–2^ and an early onset potential of 0.2 V against the reversible hydrogen electrode (RHE) for oxygen evolution.^17^ The photoanode was wired to a commercial TEG (Kimilar Peltier Cooler, TEC1-12706, 40 × 40 × 4 mm^3^, proprietary TE legs), to demonstrate the versatility of our approach. A custom setup was designed to induce a temperature difference between the two sides of a TEG (Figure 1, Figures S2–S4). Accordingly, the TEG was sandwiched between a PEC front reactor and a 3D-printed water bath acting as the heat sink, with thermal grease enabling appropriate heat transfer (Figure S3a–c). A small volume of the PEC reactor ensured a relatively rapid heating of the electrolyte solution (13.5 mL) under concentrated irradiation, whereas effective cooling was achieved by taking advantage of the high thermal conductivity of water (∼24 times higher compared to air).^18^ Variable solar concentration was achieved using a Fresnel lens, with the light intensity calibrated to 1–5 sun (100–500 mW cm^–2^) with an optical power meter (details in Supporting Information).

In this configuration, a patterned glass slide containing two FTO stripes acts as the back window of the PEC reactor (Figure 1a,c, Figure S5a). A 6 cm^2^ BiVO_4_ photoanode with a spin-coated TiCo O_2_ evolution catalyst (OEC) was deposited on one stripe following reported procedures,^19,20^ whereas Pt was selectively sputtered as the H_2_ evolution catalyst (HEC) on the second FTO stripe (see Methods in Supporting Information). The two side-by-side electrodes operated in a 0.1 M potassium borate buffer (KB_i_) solution of pH 8.5, with aqueous 0.1 M K_2_SO_4_ as electrolyte solution. The BiVO_4_ photoanode utilized short-wavelength light for oxygen evolution (<500 nm),^20^ whereas longer wavelength photons (>500 nm) were converted to heat using a matte black absorbing layer on the rear side of the glass substrate (Figures S6 and S7a,c,d). The formation of the BiVO_4_ nanoporous structure was further confirmed by scanning electron microscopy (SEM) images and X-ray diffraction (XRD) of the electrode (Figures S8a and S9).

**Figure 2:**
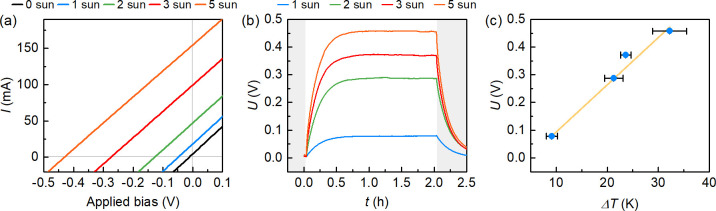
Voltage output of a TEG caused by reactor heating under light concentration. (a) Averaged *I*–*V* curves of a TE module under 1–5 sun irradiation (forward and backward scans are given in Figure S14). (b) Chronopotentiometry of the corresponding TEG under open-circuit conditions. The TEG is integrated in a Pt-TE-BiVO_4_ assembly to emulate the heating behavior observed under operation. (c) Linear relationship between the Seebeck voltage (*U*) and the temperature difference (see values in Table S1).

**Figure 3:**
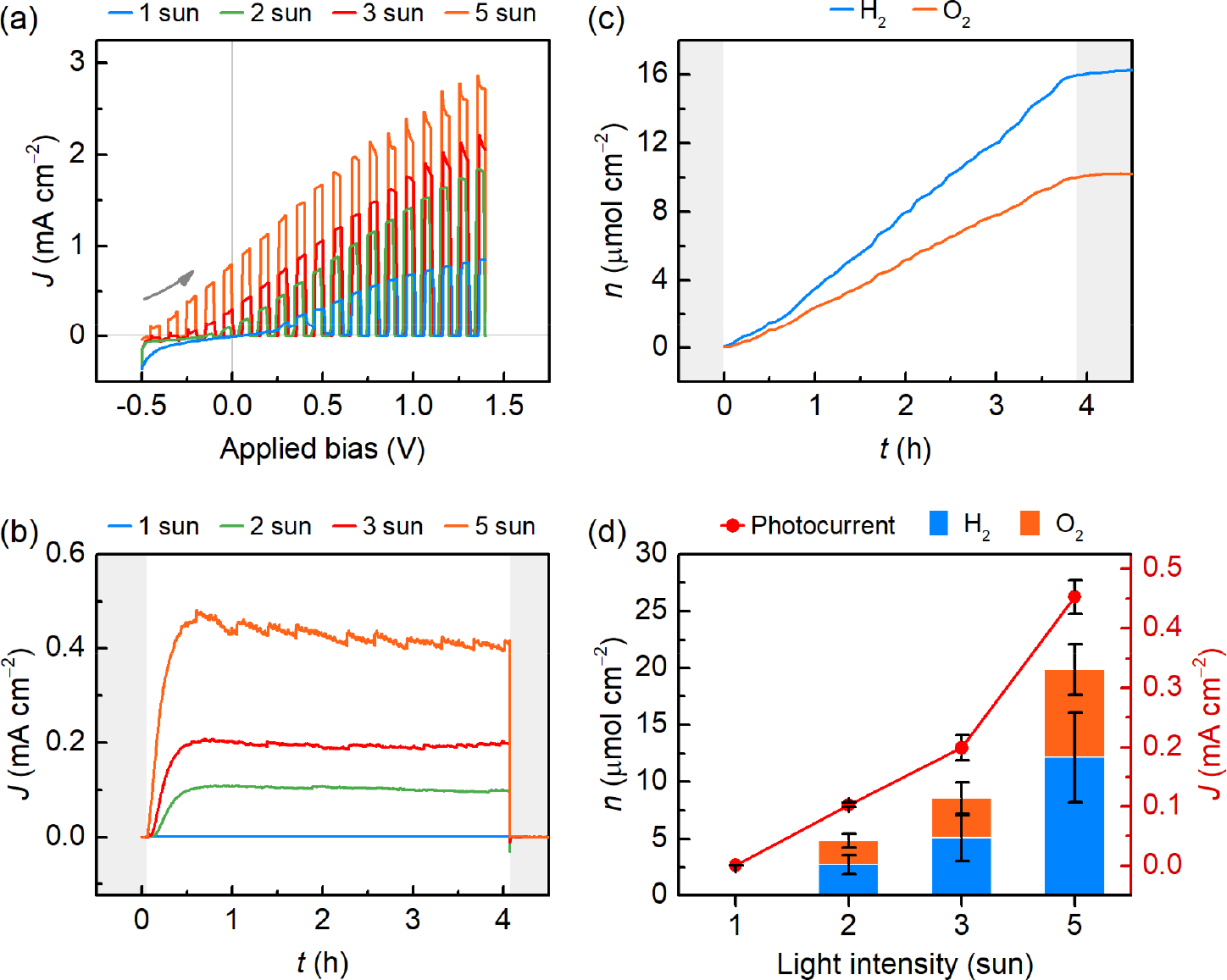
Performance of a Pt-TE-BiVO_4_ system for overall solar water splitting. (a) Chopped-light LSV of the integrated Pt-TE-BiVO_4_ system under different light intensities. (b) Representative CPE traces of bias-free water splitting under 1–5 sun irradiation. (c) Example of product evolution during 4 h CPE (Pt-TE-BiVO_4_, 5 sun). Products are quantified by gas chromatography under N_2_ flow, by sampling from the reactor headspace every 4.25 min (see Methods in Supporting Information). (d) Average steady-state photocurrents and corresponding product amounts over 4 h CPE under varying light concentration. Experiments were performed in a 0.1 M KB_i_, 0.1 M K_2_SO_4_ electrolyte solution (pH 8.5, temperature 34–61 °C), whereas the water bath was maintained at 25 °C. Gray shades indicate no irradiation.

The BiVO_4_ photoanode maintained an onset voltage of ∼0.2 V for water splitting against the Pt cathode (Pt-BiVO_4_) under concentrated light irradiation (Figure S10), whereas the electrolyte temperature reached ∼60 °C under 5 sun irradiation (Figure S11 and Table S1). While an increase in the electrolyte temperature does not significantly affect catalysis (see controls in Figures S12 and S13), the resulting temperature difference could be utilized by the TE module to provide an additional voltage of 17.1 ± 1.0 mV K^–1^. The Seebeck voltage increased linearly with the temperature difference reaching 0.46 V under open-circuit conditions, under a Δ*T* of 32.2 ± 3.3 K at 5 sun irradiation (Figure 2, Figure S14, and Table S1). Accordingly, the linear sweep voltammetry (LSV) traces of the integrated Pt-TE-BiVO_4_ system could be shifted negatively by up to ∼0.5 V under 5 sun irradiation, enabling unassisted water splitting at light intensities as low as 2 sun (Figure 3a and Figure S15).

These findings were confirmed by controlled potential electrolysis (CPE) measurements at zero applied bias voltage. The photocurrent densities stabilized within the first hour of irradiation following the change in the Seebeck voltage, as the temperature gradient reached steady state (Figures 2b and 3b). Accordingly, a photocurrent of 0.10 ± 0.01 mA cm^–2^ was observed under 2 sun irradiation, which could be further increased to 0.45 ± 0.03 mA cm^–2^ under 5 sun illumination (Figure 3b). The latter corresponded to a solar-to-hydrogen (STH) efficiency of 0.11%, which compares well among most single light absorber systems reported for overall water splitting (see Tables S2 and S3).^21^ A similar trend was observed in the product amounts, reaching 12.16 ± 3.94 μmol cm^–2^ H_2_ and 7.72 ± 2.24 μmol cm^–2^ O_2_ over 4 h of CPE under 5 sun (Figure 3c,d), whereas no products could be detected in the absence of the TE module. A deviation in the H_2_:O_2_ ratio from 2:1 was observed for most samples (Figure 3c) and can be attributed to bubble trapping and calibration limitations at low O_2_ amounts.

### TEG Integration with Perovskite-BiVO_4_ Tandem Devices

The TE element was further integrated into a perovskite-BiVO_4_ tandem structure^17,19^ to assess its potential toward enhancing the performance of established solar fuel systems. For this purpose, a triple cation mixed halide perovskite solar cell (abbreviated PVK) was sandwiched between the BiVO_4_ photoanode and the TEG (Figure 1a). The 6 cm^2^ PV cell was wired in series with both items, providing an additional photovoltage of ∼1.05 V, as shown from the histograms and *J*–*V* curves in Figures S16 and S17. The solar cell was encapsulated with a hydrophobic graphite epoxy paste covered by a flat copper foil to ensure a good thermal contact with the TEG underneath (Figure S18).

**Figure 4:**
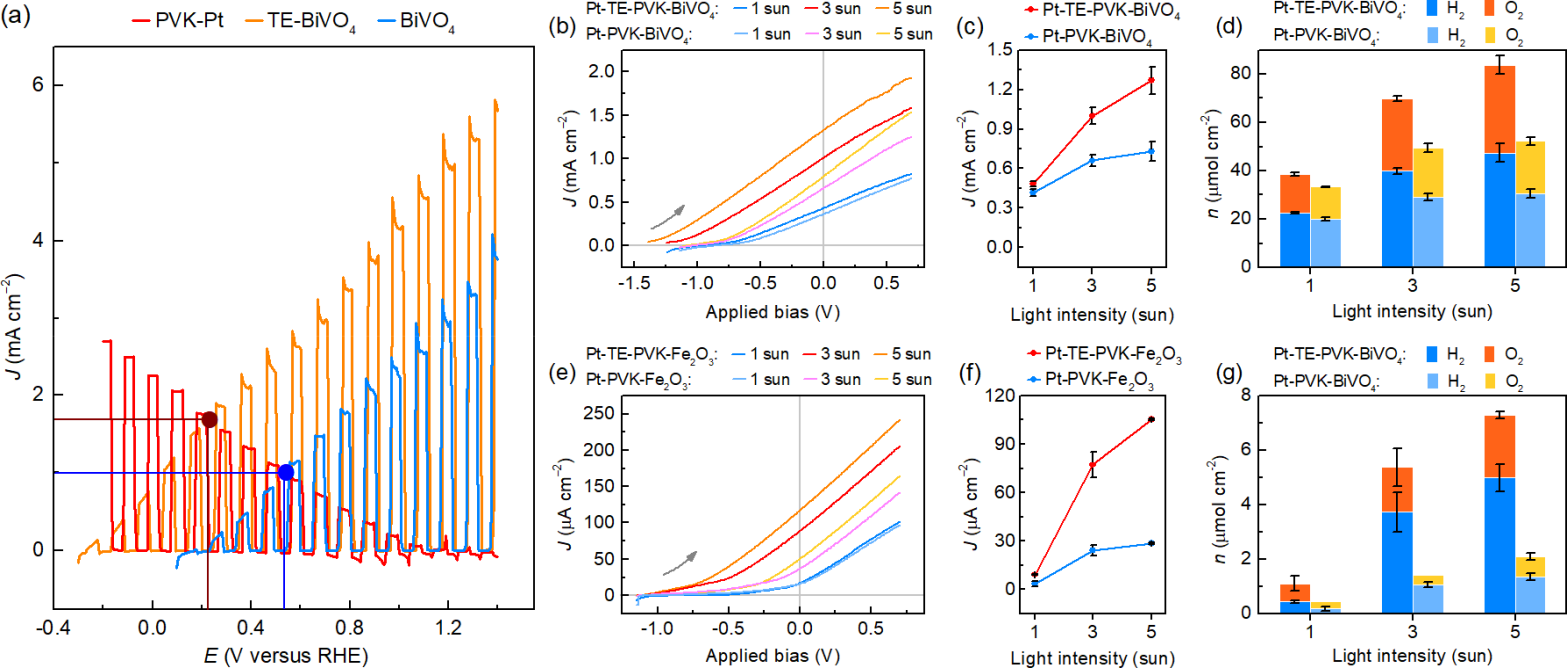
TE integration enhances the performance of PVK-oxide tandem devices for solar water splitting. (a) Intersection of LSV curves recorded for PVK-Pt, TE-BiVO_4_, and BiVO_4_ in a three-electrode setup under 5 sun irradiation. (b–d) Performance of Pt-PVK-BiVO_4_ tandem devices with or without an integrated TEG under varying light concentration. (e–g) Corresponding data for Pt-PVK-Fe_2_O_3_ and Pt-TE-PVK-Fe_2_O_3_ assemblies. (b, e) LSV traces. (c, f) Steady-state photocurrents for unassisted water splitting. (d, g) Product amounts obtained after 4 h of CPE at 0 V applied bias. Experiments were performed in 0.1 M KB_i_, 0.1 M K_2_SO_4_ buffer, pH 8.5 for BiVO_4_ and in 1 M NaOH when using a Fe_2_O_3_ photoanode (34–61 °C solution temperature; 25 °C water bath temperature).

The operating parameters of an unassisted PEC tandem device can be estimated from the overlap of the LSV curves for both photoelectrodes.^1^ The corresponding photocurrent density (*J*_op_) and applied potential (*V*_op_) help predict the product rates and selectivity, which become key in cases when a high overpotential must be overcome, for instance in unassisted CO_2_ reduction.^22^ The integrated TE element holds promise to improve both parameters, by shifting the intersection point of the two electrodes. To illustrate this, we plotted the LSV curves of the BiVO_4_ photoanode wired to the TEG (TE-BiVO_4_) and of the perovskite PV cell wired to the Pt cathode (PVK-Pt, equivalent to our buried PV photocathodes^17,19^), which were determined in a three-electrode configuration with a Ag/AgCl reference and Pt counter electrode. LSV curves recorded under 1 and 5 sun irradiation displayed indeed that not only is the *J*_op_ expected to be improved, but also the *V*_op_ could be altered (Figure 4a and Figure S19).

LSV curves of the full Pt-TE-PVK-BiVO_4_ system confirmed the beneficial effect of TEG integration, which became again more significant under elevated light flux. In this case, the photocurrent matching between the BiVO_4_ and PVK-Pt elements governs the overall tandem performance. Both light absorbers attain matching photocurrent densities of a few mA cm^–2^ (Figure 4a), which overlap at an absolute photocurrent of around 6 mA under 5 sun illumination (Figure S19c). In contrast, the TE element can operate at currents above 100 mA under a similar light concentration, due to its minimal electrical resistance of 2.6 Ω (see *I*–*V* curves in Figure 2a). As a result, the operating conditions of the serially connected Pt-TE-PVK-BiVO_4_ system are close to the open-circuit voltage of the TE element, maximizing the applied TE voltage without affecting the overall photocurrent (Figure S19c).^23^ Accordingly, a ∼0.5 V negative shift could be again observed under 5 sun irradiation, resulting in a notable improvement in the onset voltage of approximately −1.4 V for overall water splitting (Figure 4b). Under these conditions, the Pt-TE-PVK-BiVO_4_ system reached a steady-state photocurrent of 1.27 ± 0.10 mA cm^–2^ (STH of 0.31 ± 0.13%) yielding the highest improvement, 1.7 times, over the 0.73 ± 0.08 mA cm^–2^ photocurrent of a classical PVK-BiVO_4_ tandem device (Figure 4c, Figure S20a, Tables S4 and S5). Similar trends were observed in the amounts of products, amounting to a total of 47.5 ± 3.8 μmol cm^–2^ H_2_ and 36.3 ± 3.6 μmol cm^–2^ O_2_ for the Pt-TE-PVK-BiVO_4_ system (4 h CPE, 5 sun; Figure 4d and Figure S20b).

### Scope of the Thermoelectric Integration

While the early onset potential of BiVO_4_ toward O_2_ evolution makes it an excellent photoanode material for tandem PEC devices, the onset potentials of other established light absorbers (e.g., WO_3_, or hematite) restrict the choice of photocathodes for overall water splitting. Thermoelectric integration has the potential to overcome these limitations, by providing the additional voltage required for unassisted water splitting.

To demonstrate the versatility of our concept to a broad scope of tandem configurations, we therefore replaced BiVO_4_ with an α-Fe_2_O_3_ photoanode. Although hematite is an earth abundant photoanode material with a favorable optical bandgap (2.2 eV) and good stability under deleterious chemical conditions, it suffers from poor charge transport and short excited-state lifetime,^24^ resulting in an onset potential of only 0.8 V vs RHE for O_2_ evolution (Figure S21). Here, 6 cm^2^ α-Fe_2_O_3_ photoanodes were fabricated on FTO-coated glass substrates via a previously reported hydrothermal method.^25^ Photoanodes showed an absorption edge around 600 nm and a deep red color (Figure S6 and S7b). SEM and XRD confirmed the formation of porous α-Fe_2_O_3_ photoanodes (Figures S8b and S9).

The heat-induced ∼0.5 V shift in the onset voltage (Figure 4e) was especially beneficial for the Pt-TE-PVK-Fe_2_O_3_ system, as PVK-Pt and α-Fe_2_O_3_ electrodes only display a limited LSV overlap (Figure S22).^26,27^ The average steady-state photocurrents amounted to 9.08 ± 0.54 and 105.4 ± 0.7 μA cm^–2^ for Pt-TE-PVK-Fe_2_O_3_ devices, whereas Pt-PVK-Fe_2_O_3_ only reached 3.55 ± 1.64 and 28.5 ± 0.7 μA cm^–2^ under 1 and 5 sun, respectively. Accordingly, the TE integration yielded a 3.7 times photocurrent improvement under 5 sun, which is significantly higher than the 1.7× improvement attained in the corresponding Pt-TE-PVK-BiVO_4_ arrangement (Figure 4f, Figure S23a, Tables S6 and S7). More importantly, this represents a 29.7× improvement for Pt-TE-PVK-Fe_2_O_3_ at 5 sun over the photocurrent of Pt-PVK-Fe_2_O_3_ tandem devices under 1 sun irradiation, highlighting the beneficial complementary effects of light concentration and thermoelectric waste heat harvesting. The photocurrents began to plateau under stronger light irradiation for both BiVO_4_ and Fe_2_O_3_ systems, following the conventional *J*–*V* shape of PV and PEC components. The same trends were observed in the product evolution, amounting to 4.99 ± 0.50 μmol cm^–2^ H_2_ and 2.30 ± 0.11 μmol cm^–2^ O_2_ for Pt-TE-PVK-Fe_2_O_3_ over 4 h of CPE under 5 sun (Figure 4g and Figure S23b).

While differences in light concentration, photovoltages, and catalytic overpotentials prevent a direct correlation between the efficiency of the individual components and their assembly, these results indicate that an additional Seebeck voltage is key to enable overall solar fuel production in PEC systems with insufficient photovoltage, such as Pt-BiVO_4_ or Pt-PVK-Fe_2_O_3_. The additional voltage provided by the TE element may allow such PEC tandems to overcome the high overpotentials associated with other demanding reactions including CO_2_ reduction^22^ and N_2_ fixation, whereas the increased temperature may further improve reaction kinetics.^4,28^ In addition, thermal integration may prevent overheating or temperature fluctuations, which would avoid the degradation and photovoltage losses encountered in classical PV-electrolysis systems.

In an industrial context where the total output becomes more relevant than device efficiency, this technology could provide significant benefits in terms of costs and material consumption,^29,30^ as large-area devices may be replaced by small reactors coupled to relatively inexpensive plastic Fresnel lenses. Accordingly, light concentration would offset the cost of adding an additional TE module, making such integrated circuits more commercially competitive. As great emphasis has been recently placed on the advantages of light concentration and thermal management,^4,31^ our approach may provide a route to improve the performance of such upscaled systems. However, further design improvements are still needed with respect to heat transfer, thermal insulation, or controlled electrolyte flow to prevent overheating.^4,28^ Metal fingers will also be required to mitigate resistive losses on a large scale, as in-plane parasitic effects are already noticeable in the case of our 6 cm^2^ light absorbers, limiting photocurrent densities below those of small-scale tandem PV-PEC devices.^32^

## Conclusions

This work demonstrates that direct waste heat utilization can benefit PEC devices, as the additional thermoelectric bias voltage increases product output and device photocurrent. A steady temperature difference is maintained via passive heating across the two sides of a commercial TE module, by integrating either single or tandem light absorbers with the TEG in a single optical light path. This thermal management approach enables unassisted overall water splitting even for PEC systems with insufficient photovoltage. Accordingly, overall water splitting is achieved with a single BiVO_4_ photoanode and a Pt cathode at light intensities as low as 2 sun, whereas the ∼0.5 V shift in the onset voltage induces a 3.7× photocurrent enhancement under 5 sun for a perovskite-Fe_2_O_3_ tandem. This integrated energy harvesting system makes use of charge, light, and heat flows, providing a proof-of-concept for scalable, overall energy management.

## Supporting Information Available

Experimental details, synthesis procedures, device assembly and characterization, supplementary tables, and supplementary data figures (PDF)
