# Supplementary material for: Thermoelectric–Photoelectrochemical Water Splitting under Concentrated Solar Irradiation
Source: J Am Chem Soc. 2023 Jun 13;145(25):13709–14. doi: 10.1021/jacs.3c01892 (PMC10311528; doi:10.1021/jacs.3c01892)
Supplement: Supplementary file 1 — ja3c01892_si_001.pdf [file ja3c01892_si_001.pdf]

Supporting Information

Thermoelectric-photoelectrochemical water splitting under concentrated solar irradiation

Chanon Pornrungroj,[1,†] Virgil Andrei,[1,†] Erwin Reisner[1,*]

[1] *Yusuf Hamied Department of Chemistry, University of Cambridge, Lensfield Road, Cambridge CB2 1EW, United Kingdom.*

** Email: reisner@ch.cam.ac.uk; Web: http://www-reisner.ch.cam.ac.uk.*

[†] These authors contributed equally.

Methods

Materials. FTO-coated glass (~7 Ω sq^{-1}, Sigma-Aldrich), H_2SO_4 (>95%, Fisher), H_2O_2 (>30% w/v, Fisher), Zn (dust, 98+%, ACROS), HCl (fuming, 36.5-38%, Honeywell), $Ni(NO_3)_2 \cdot 6\,H_2O$ (≥98.5%, Sigma-Aldrich), ethylene glycol (99.8%, anhydrous, ACROS), ethylenediamine (absolute, ≥99.5%, Fluka), PbI_2 (99.99%, trace metals basis, TCI), $PbBr_2$ (for Perovskite precursor, TCI), formamidinium iodide (Dyesol), methylammonium bromide (Dyesol), N,N-dimethylformamide (anhydrous, 99.8%, Sigma-Aldrich), 1-methyl-2-pyrrolidone (99.5%, extra dry over molecular sieves, ACROS), dimethyl sulfoxide (ACS reagent, ≥99.9%), chloroform (99.9%, extra dry over molecular sieves, stabilized, ACROS), matt black spray paint (Simply Sprays), thermal grease CTCM78-1 (7.8 W m^{-1} K^{-1}, Conrad), [6,6]-phenyl C_{61} butyric acid methyl ester (PCBM, >99%, Ossila), chlorobenzene (extra dry over molecular sieves ≥99.5%, ACROS), polyethylenimine (80% ethoxylated solution, 35-40 wt.% in H_2O, average MW 70,000, Sigma-Aldrich), 2-propanol (≥99.5%, Honeywell), poly[bis(4-phenyl)(2,4,6-trimethylphenyl)amine] (PTAA, MW 17,700, EM INDEX), F4TCNQ (97%, Sigma-Aldrich), graphite powder (<20 µm, Sigma-Aldrich), $Bi(NO_3)_3 \cdot 5H_2O$ (98%, Sigma-Aldrich), Cu foil (1.0 mm, 99.99% metal basis, Fisher), Araldite 5-Minute Rapid two component epoxy, Araldite Standard two component epoxy, copper wire (0.25 mm diameter, 99.999%, Sigma-Aldrich), NaI (laboratory reagent grade, Fischer Scientific), p-benzoquinone (≥98%, Sigma-Aldrich), ethanol (absolute, VWR), vanadyl acetylacetonate (≥97.0%, Fluka), dimethyl sulfoxide (99+%, Alfa Aesar), NaOH (analytical reagent grade, Fisher), ferric chloride (≥99.9%, Sigma-Aldrich) and sodium nitrate (≥99.0%, Sigma-Aldrich) were used without further purification unless otherwise stated.

Preparation of 6 cm^2 $BiVO_4$ and α-Fe_2O_3 photoanodes, and Pt counter electrode. 6 × 4 cm^2 FTO-coated glass slides were selectively etched with Zn dust and 2 M aqueous HCl to provide an active area of ~2×3 cm^2 for $BiVO_4$ or α-Fe_2O_3, and 0.5×3 cm^2 for Pt (Supplementary Fig. 5a), before cleaning the glass slides in Piranha solution. The 6 cm^2 $BiVO_4$ electrodes were prepared following previously reported protocols.[1,2] In brief, a first solution was prepared by dissolving $Bi(NO_3)_3$ (0.194 g, 0.4 mmol) and NaI (1.199 g, 8.0 mmol) in Milli-Q water (20 mL) using an ultrasonic probe (Fischer Scientific Model 120 Sonic Dismembrator) for 3 min. After that, the pH was adjusted to 1.20 using concentrated nitric acid. A second solution consisting of benzoquinone (0.292 g, 2.7 mmol, 0.3 M) in absolute ethanol (9 mL) was also sonicated for 3 min. The two solutions were mixed and stirred for 30 min at room temperature to obtain a dark brown BiOI precursor solution. The orange BiOI layer was then

electrodeposited onto the active area of the FTO slides, by maintaining a potential of -0.3 V against a Ag/AgCl reference electrode for 5 s, followed by -0.1 V for 180 s. A vanadyl acetyl acetonate (VO(acac)$_2$) solution was prepared by sonicating VO(acac)$_2$ (0.530 g, 2.0 mmol) in 5 mL DMSO for 5 min. 40 µL cm^{-2} of the VO(acac)$_2$ solution was drop-casted onto the BiOI active areas, before heating the FTO slides at 723 K for 60 min, with a ramp rate of 1 K min^{-1}. After the glass slides were left to cool down to room temperature, a NaOH (0.4 M) aqueous solution was used to wash the brownish V_2O_5 crust from the surface resulting in a bright yellow $BiVO_4$ photoanode. A [$Ti_4O(OEt)_{15}(CoCl)$] precursor was synthesized and spin-coated as previously reported, to form a $TiCoO_x$ (TiCo) O_2 evolution catalyst.[3]
Fe_2O_3 was prepared by a hydrothermal procedure.[4] In brief, 0.09 M $FeCl_3$ and 0.1 M $NaNO_3$ were dissolved in Milli-Q water. The pH was adjusted to 1.4 using 1 M HCl and 50 mL of the solution was transferred to a 200 mL Teflon-lined stainless steel autoclave. A washed FTO glass slide was then immersed in the solution and heated at 95 °C for 4 h. The obtained yellow film was then calcinated at 550 °C for 2 h and further annealed at 800 °C for 20 min. Finally, a 5 nm thick Pt film was deposited by magnetron sputtering onto the previously etched 0.5×3 cm^2 FTO to form the hydrogen evolution counter electrode. For the Pt-TE-$BiVO_4$ and Pt-TE-Fe_2O_3 experiments, a black absorbing layer was spray painted on the rear side of the glass substrate.

Preparation of 6 cm^2 PVK. The inverse-structure perovskite cells were prepared according to previously reported procedures with few modifications.[5] In brief, 3.5×4 cm^2 FTO glass slides were selectively etched with Zn dust and 2 M HCl to provide an active area of ~2×3 cm^2 (Figure S5b). A NiO_x hole transport layer (HTL) was first uniformly deposited onto the FTO substrate via spin-coating ~0.3 mL of a 1.0 M $Ni(NO_3)\cdot 6H_2O$, 1.0 M ethylenediamine solution in ethylene glycol, followed by annealing at 573 K. PTAA doped with F4TCNQ solution (0.3 mL) was spin coated onto the FTO|NiO_x substrate inside a N_2-filled glovebox as a second HTL. A cesium formamidinium methylammonium (CsFAMA) perovskite precursor solution was prepared by adding 96 µL of 1.5 M CsI in DMSO stock solution to 2000 µL of $FAMA_{0.22}Pb_{1.32}I_{3.2}Br_{0.66}$ solution in DMF (in 1020µL), DMSO (680 µL), and NMP (300 µL). A smooth perovskite layer was then deposited by spin coating 0.3 mL of the precursor solution in a two-step procedure, first 10 s at 1000 rpm and then 35 s at 6000 rpm, using 0.6 mL chloroform as the antisolvent ~7 s before the end. The perovskite layer was then annealed at 373 K for 30 min. A thin PCBM layer was deposited as ETL by spin coating ~0.2 mL of a 35 mg mL^{-1} PCBM solution in chlorobenzene at 3000 rpm for 45 s. Next, a 3.87 µL mL^{-1} PEIE solution in isopropanol (0.4 mL) was spin coated under ambient conditions at 3000 rpm for

30 s, before storing the samples under inert atmosphere. Lastly, a 100 nm silver layer was evaporated through a custom-made mask to form the top electrical contact.

The inverse-structure perovskite cells were then encapsulated with graphite epoxy (GE) paste and a copper foil to form PVK. Graphite powder was mixed with Araldite Standard two component epoxy in 3:4 mass ratio to create a GE paste.[6] The paste was evenly spread on top of the Ag contact layer of the PV device. The copper foil (1.0 mm thick, 4×4 cm^2) was then slightly pressed against the paste to form an electrical contact. The GE was allowed to settle for 24 hours. Finally, Araldite 5-Minute Rapid two component epoxy was used to seal the edges and left to settle overnight (Figure S18).

Setup assembly. A TE module is first attached to the aluminium wall of a 3D-printed water reservoir heat sink using thermal paste. The water reservoir is equipped with an additional cooling block, which connects to a chiller, to maintain a steady water bath temperature (25 °C) on the cold side of the TE module under concentrated irradiation (Figure S4). The same thermal paste is used to interface the bottom copper contact of the PVK to the hot side of the TE module. A $BiVO_4$ photoanode is next placed on top of the perovskite PV cell. The PEEK reactor is next placed over the TE-PVK-$BiVO_4$ stack and a lid tightens all components in place. The $BiVO_4$ photoanode is connected in series to a PVK, the TE module, and Pt cathode. In case of the Pt-TE-$BiVO_4$ and Pt-TE-Fe_2O_3 arrangements, PVK was not used (Figure S3).

Photoelectrochemical characterization. Perovskite solar cells were characterized under air, without masking, using a Keithley 2635 source meter and an Abet Technologies Sun 2000 Solar Simulator, which was calibrated with a certified RS-OD4 reference silicon diode. For the PEC setup, a LOT-QD LS0816-H large area solar simulator was employed with an Air Mass 1.5 Global (AM 1.5G) solar filter. The solar concentration was achieved with a commercial Fresnel lens (XL Full Page, 3× magnification). The light intensity was set to 100-500 mW cm^{-2} (i.e. 1-5 sun) with a certified Newport 843-R optical power meter, by adjusting the distance between lens and PEC reactor. The electrolyte temperature was determined using a thermometer probe submerged in the PEC reactor. (Photo)electrochemical tests were conducted in a 0.1 M potassium borate buffer (KB_i) solution, which contained 0.1 M K_2SO_4 (adjusted to pH 8.50 with 10 M KOH) for experiments with $BiVO_4$ photoelectrodes, while 1 M NaOH was used for experiments with Fe_2O_3. Experiments were conducted on an Ivium CompactStat.e potentiostat in a two-electrode configuration, with the photoanode and sputtered platinum cathode placed side-by-side. All the measurements were conducted in a custom made one-compartment PEC cell, under stirring (see Figures S3 and S4). Prior to the electrochemical

measurements, the cell was sealed with rubber septa and the solution was purged with N_2 for 30 min. All purging needle holes were then sealed with Loctite superglue Universal adhesive. Cyclic voltammetry scans were recorded between −0.5 and 1.3 V for the TE-$BiVO_4$, −1.4 and 0.7 V for the TE-PVK-$BiVO_4$, and -1.15 and 0.7 V for the TE-PVK-Fe_2O_3 (10 mV s^{-1} scan rate). The STH efficiency of this system was calculated using Equation (S1), where J_{0V} is the bias-free photocurrent density, FY is Faradaic yield, and P_{total} is the total light intensity flux (100-500 mW cm^{-2}).[1]

$$\mathrm{STH} = \left[\frac{|J_{0V}|\times(1.23\mathrm{V})\times\mathrm{FY}}{\mathrm{P_{total}}}\right] \quad \text{(S1)}$$

H_2 and O_2 quantification were performed in flow by automated gas sampling every 4.25 min from the headspace of the reactor. The reactor was continuously flushed with N_2 at a flow rate of 5.0 sccm using a Brooks GF040 mass flow controller. The composition of the gas mixture was analyzed using a Shimadzu GC-2010 Plus gas chromatograph with a barrier discharge ionization detector.[7] An Alicat M–Series mass flow meter was employed to check the flow rate at the GC outlet.

Material Characterization. A TESCAN MIRA3 FEG-SEM instrument with an Oxford Instruments Aztec Energy X-maxN 80 system was used to collect the SEM images and EDX mapping. X-ray diffraction patterns of the samples were measured with a PANalytical Empyrean Series 2 instrument using a Cu Kα source operated at 40 kV and 40 mA. UV-vis diffuse reflectance spectra (DRS) were recorded on a Varian Cary 50 Bio Spectrophotometer with a Harrick Scientific Video Barrelino probe.

Statistics. Average values are calculated from sample triplicates except for Pt-TE-PVK-Fe_2O_3 and Pt-PVK-Fe_2O_3, where the values from duplicates were reported. The reported errors correspond to the standard deviation.

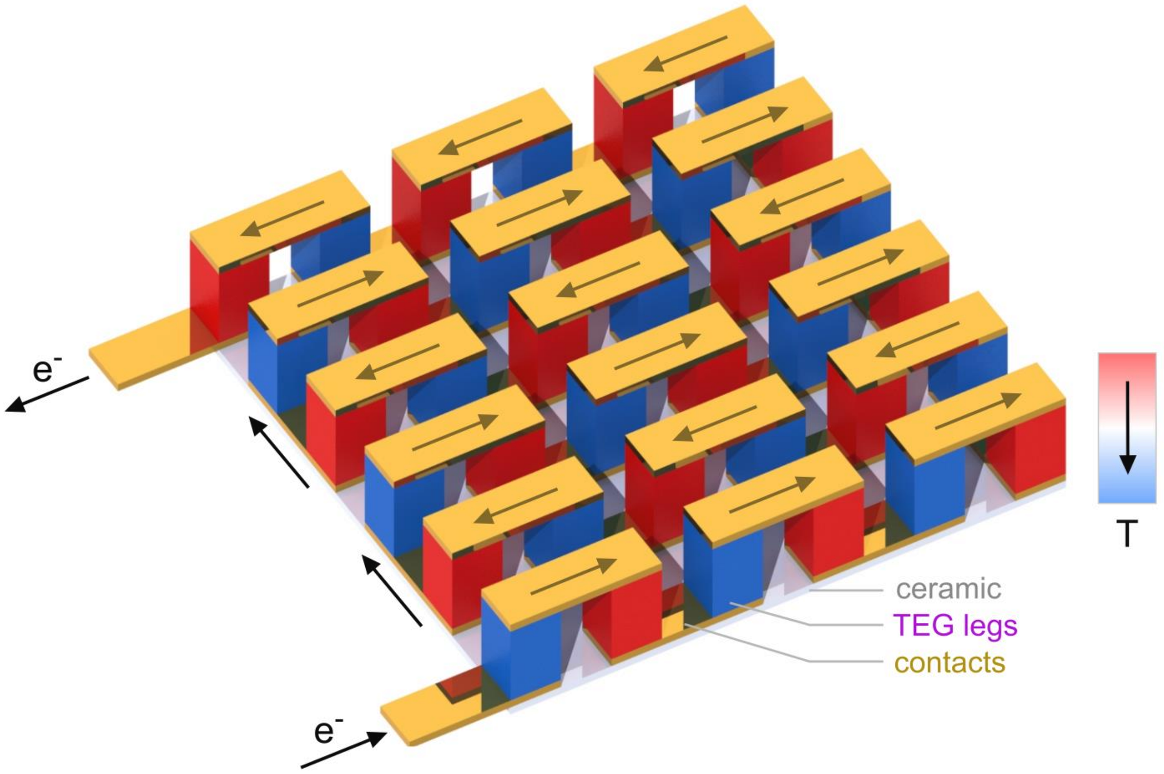

Figure S1. Schematic representation of a thermoelectric generator (TEG). The top ceramic plate is removed to reveal the thermoelectric legs. Pairs of p- and n-type legs induce a positive or negative Seebeck voltage when subjected to a temperature difference. The legs are assembled thermally in parallel and electrically in series, so the individual contributions sum up to a larger voltage difference.

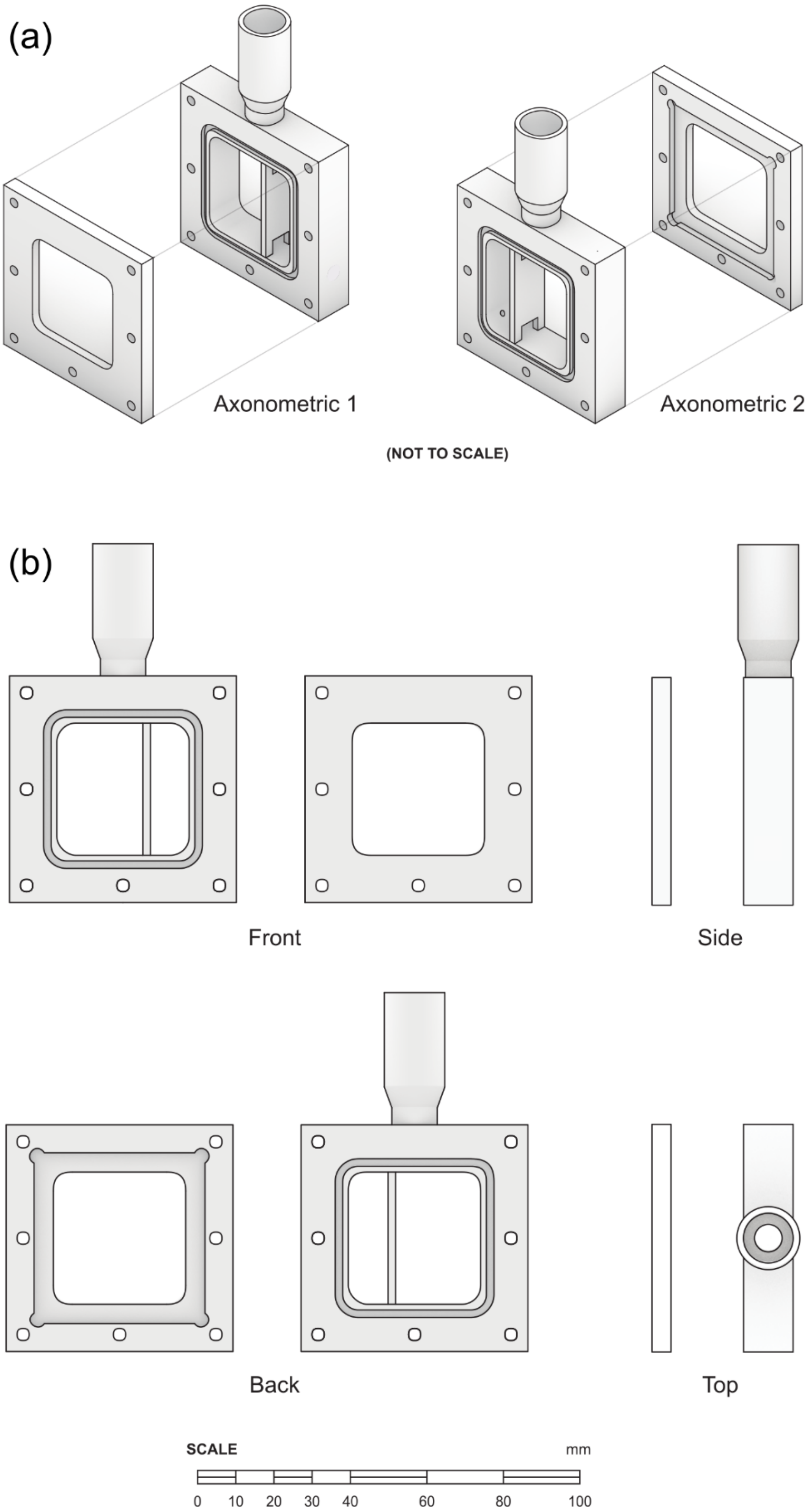

Figure S2. Sketches of the custom PEC reactor and its components. (a) Perspective views. (b) 2D projections. The PEEK-machined reactor can accommodate samples with photoactive areas of up to 3.5×3.5 cm^2 and covers a total area of 6.0×6.0 cm^2. A thin plate mitigates the electrolyte mixture between the cathodic and anodic compartments, preventing O_2 reduction on the cathodic side. The plate presents a hole on the top side to enable simultaneous gas quantification in flow, and a second opening at the bottom to prevent the build-up of a pH difference.

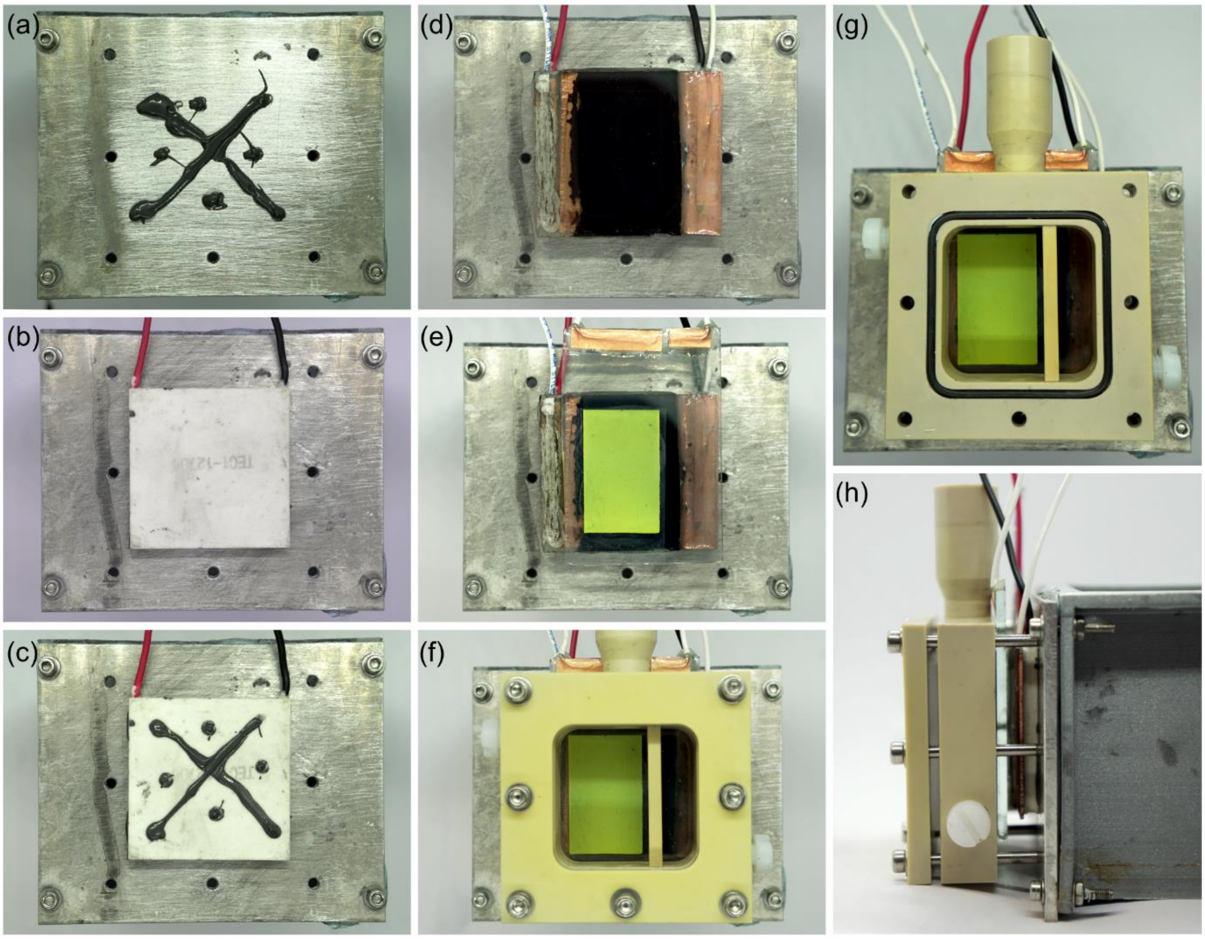

Figure S3. Step-by-step assembly of the Pt-TE-PVK-$BiVO_4$ device. (a, b) A thermoelectric (TE) module is first attached to the aluminium wall of a 3D-printed water reservoir using thermal paste. The water reservoir acts as a heat sink, maintaining a steady temperature on the cold side of the TE module. (c, d) The same thermal paste is used to interface the bottom copper contact of the PV device to the hot side of the TE module. (e) A $BiVO_4$ photoanode is next placed on top of the perovskite PV cell. (f-h) The PEEK reactor is next placed over the Pt-TE-PVK-$BiVO_4$ stack (g), and a lid tightens all components in place (f, h). Step (d) is skipped in case of the Pt-TE-$BiVO_4$ and Pt-TE-Fe_2O_3 arrangements.

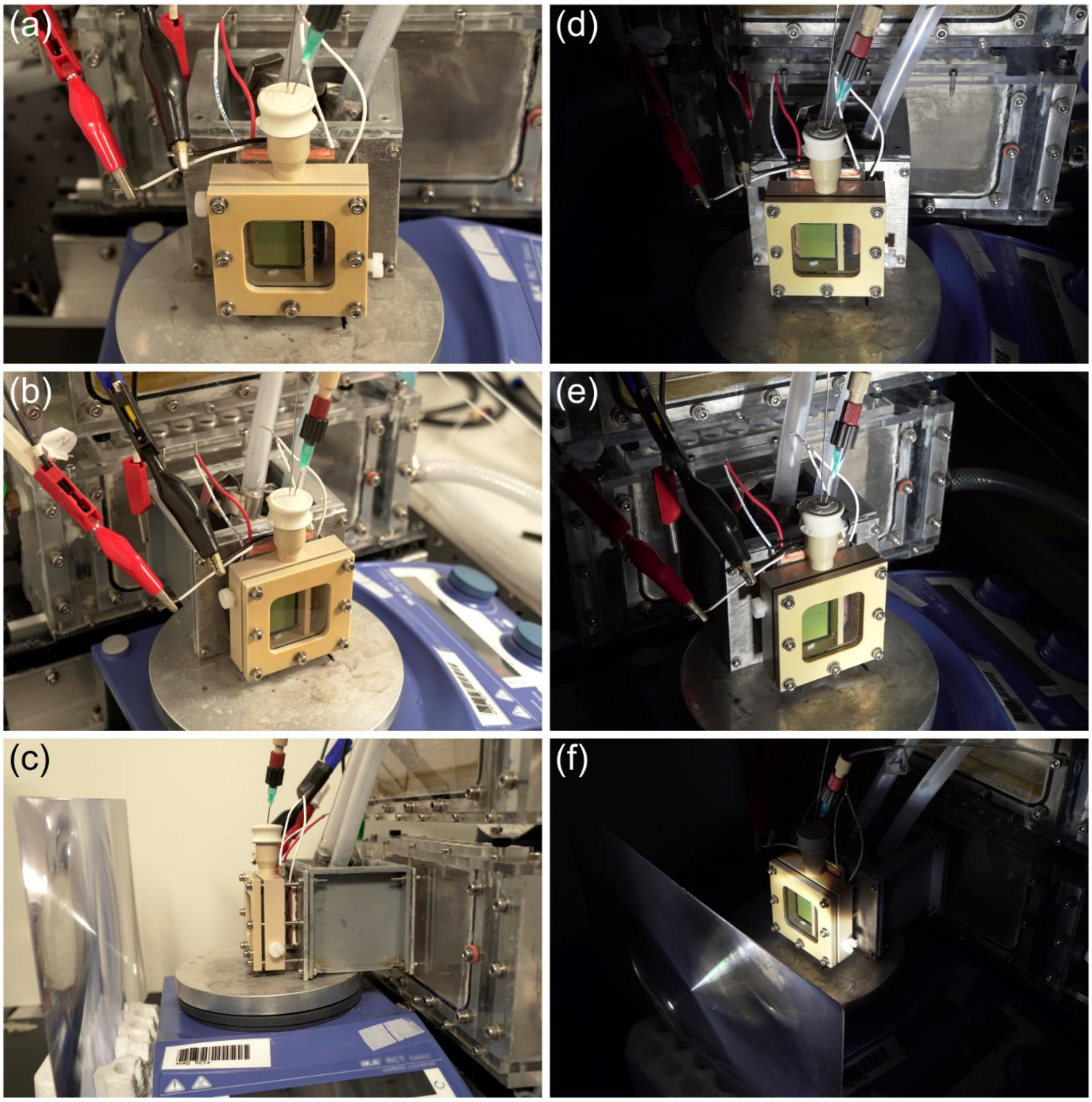

Figure S4. Photographs of the TE-PVK-$BiVO_4$ reactor under operation. (a-c) Depictions of the assembly without irradiation. (d, e) The reactor is operated under 1 sun illumination. (f) The reactor performs water splitting under concentrated light irradiation, achieved via a Fresnel lens. Product quantification occurs under N_2 flow. An additional cooling block is mounted inside the 3D-printed reservoir, to maintain a steady water bath temperature under concentrated irradiation.

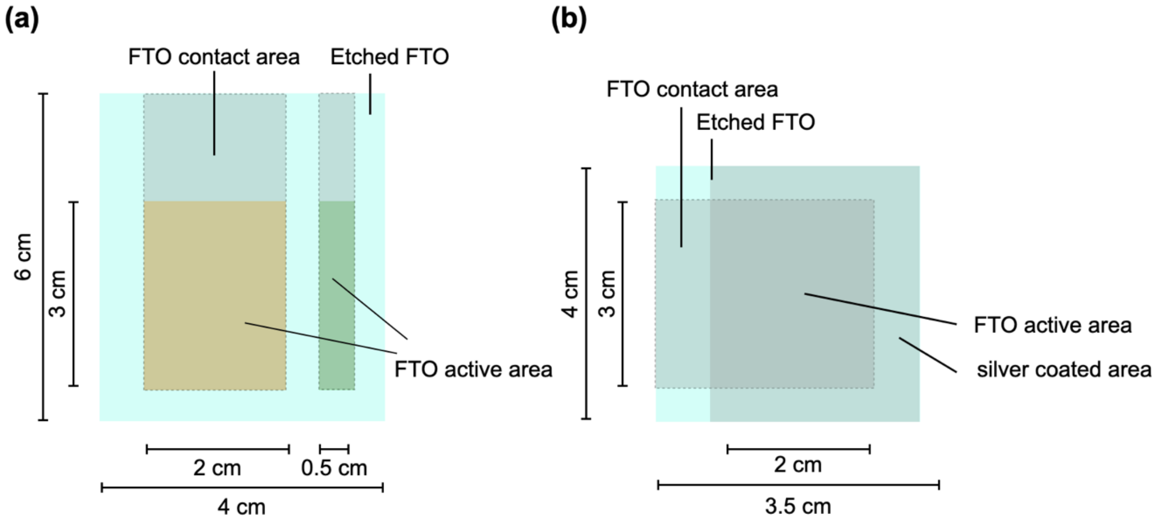

Figure S5. Etching patterns on FTO glass substrates, defining the active and contact areas for photoanodes and perovskite cells. (a) Two separate stripes of FTO are patterned on a glass substrate, providing active areas of 6 cm^2 and 1.5 cm^2 for the $BiVO_4$ or Fe_2O_3 photoanode, and the Pt H_2 evolution cathode, respectively. (b) The photoactive area of the perovskite PV cell is defined by the overlap between the FTO stripe and the evaporated Ag layer, amounting to 6 cm^2.

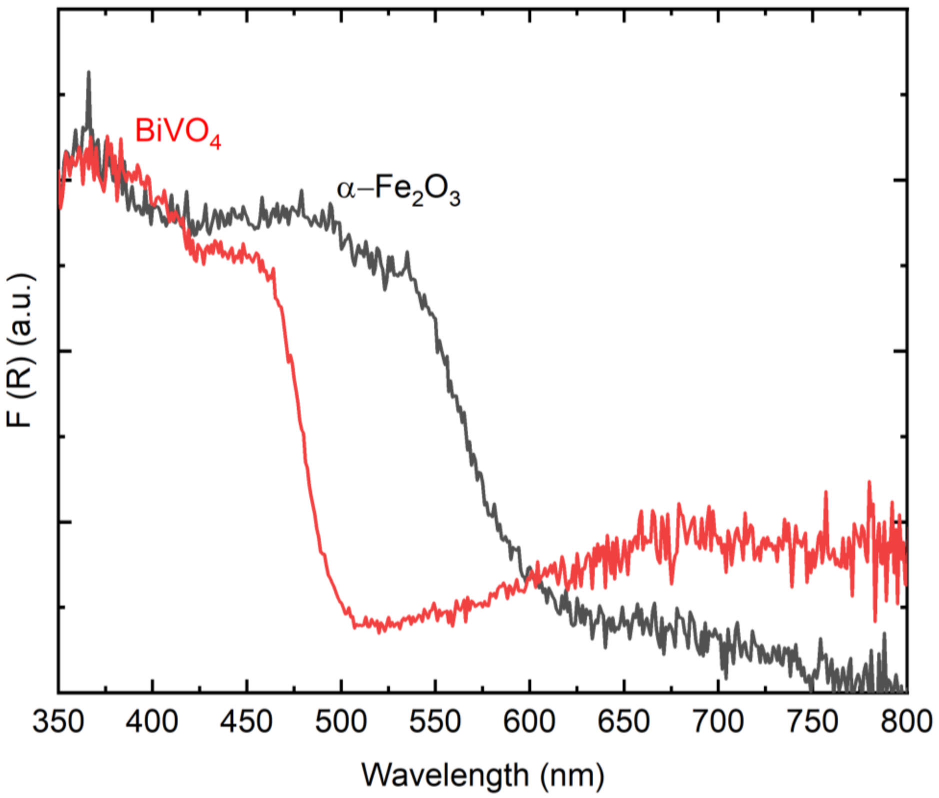

Figure S6. UV-Vis diffuse-reflectance spectra for $BiVO_4$ and Fe_2O_3 photoanodes. $BiVO_4$ shows an absorption onset at 500 nm, consistent with monoclinic $BiVO_4$.[8] Fe_2O_3 exhibits a characteristic absorption edge at around 600 nm.[4]

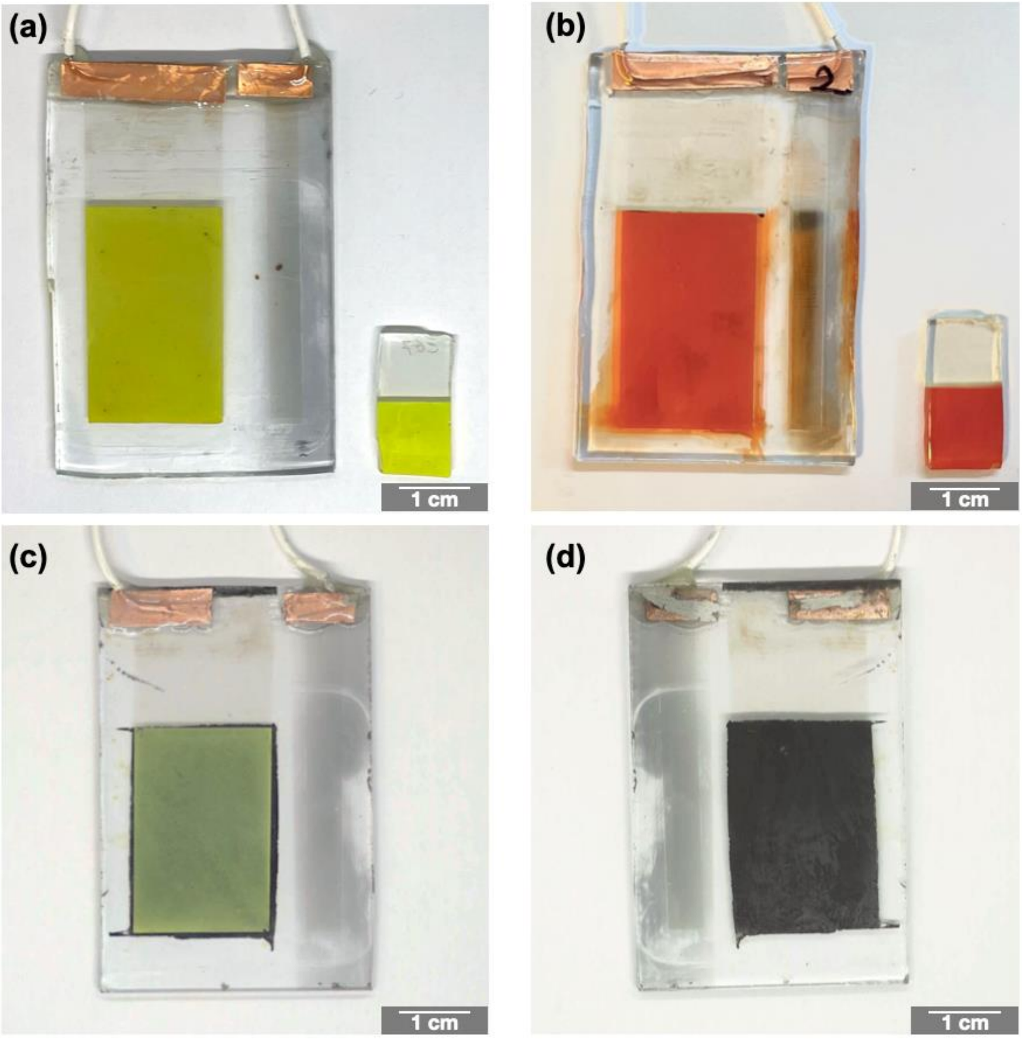

Figure S7. Photographs of a patterned glass substrate containing a $BiVO_4$ or Fe_2O_3 photoanode and the Pt H_2 evolution cathode. (a) 2×3 cm^2 (left) and 1×1 cm^2 (right) $BiVO_4$ photoanode with a spin-coated TiCo O_2 evolution catalyst. (b) 2×3 cm^2 (left) and 1×1 cm^2 (right) Fe_2O_3 photoanode. (c,d) Front (c) and back side (d) view of the $BiVO_4$ photoanode with spray-painted matte black absorbing layer.

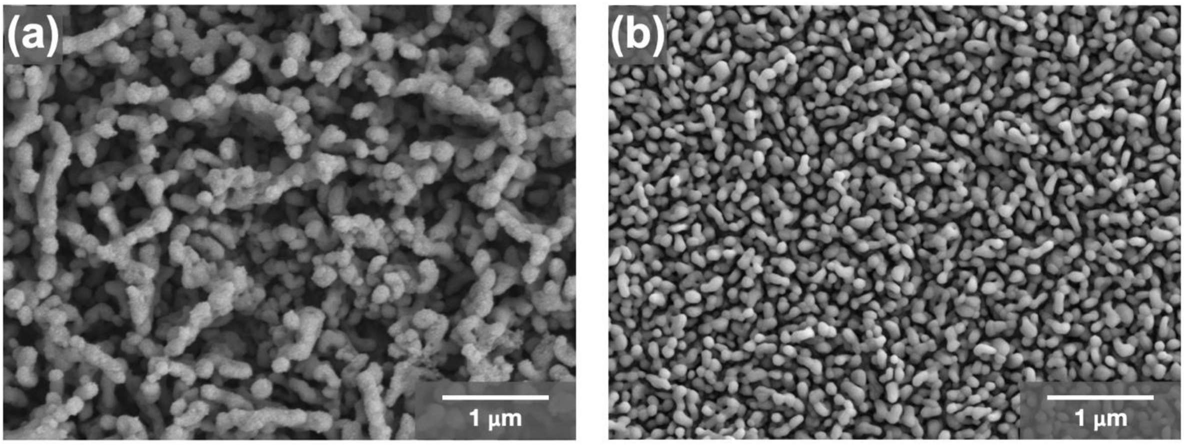

Figure S8. Scanning electron microscopy (SEM) images of the photoanodes. (a) $BiVO_4$ photoanode with a spin-coated TiCo O_2 evolution catalyst. (b) Fe_2O_3 photoanode.

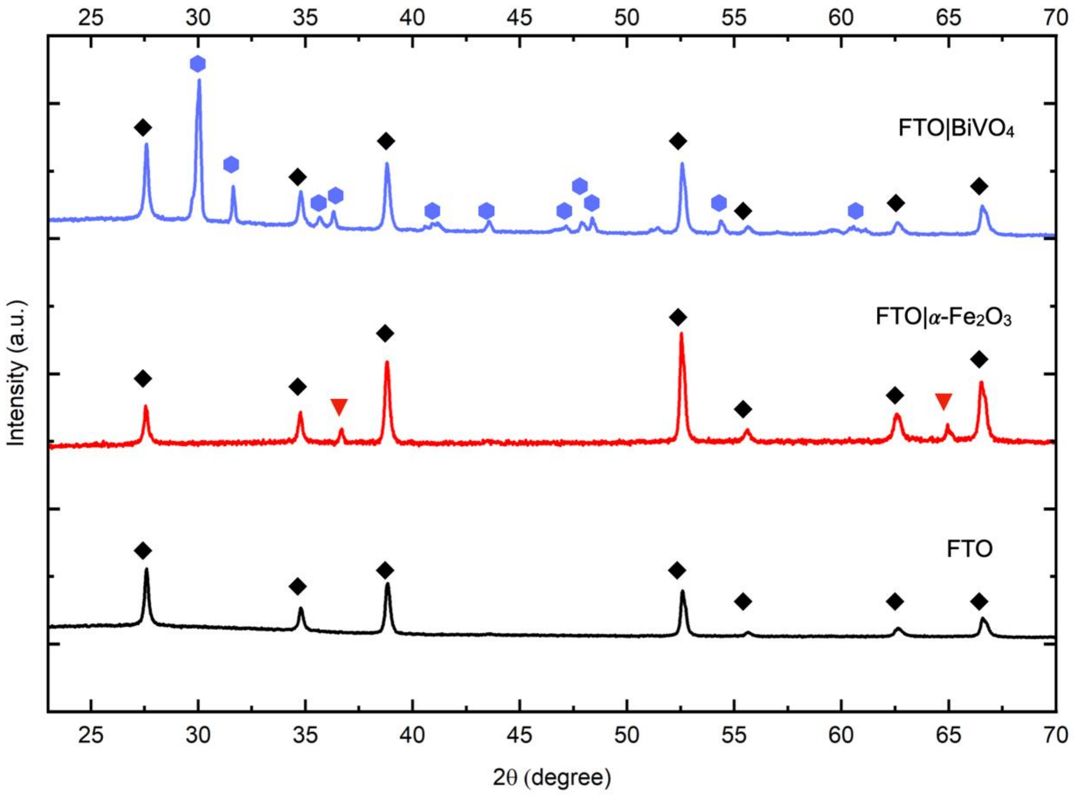

Figure S9. X-ray diffraction (XRD) patterns for FTO|$BiVO_4$ and FTO|α-Fe_2O_3. The black diamonds, red triangles and blue hexagons indicate the diffraction patterns of FTO, α-Fe_2O_3, and $BiVO_4$, respectively. The peaks located at 35.6 and 64.0 are indexed to the (110) and (300) lattice diffraction of α-Fe_2O_3 (PDF No. 00-033-0664). XRD peaks of $BiVO_4$ match with the standard monoclinic scheelite phase of $BiVO_4$ (PDF No. 00-014-0688); no other phases were found.

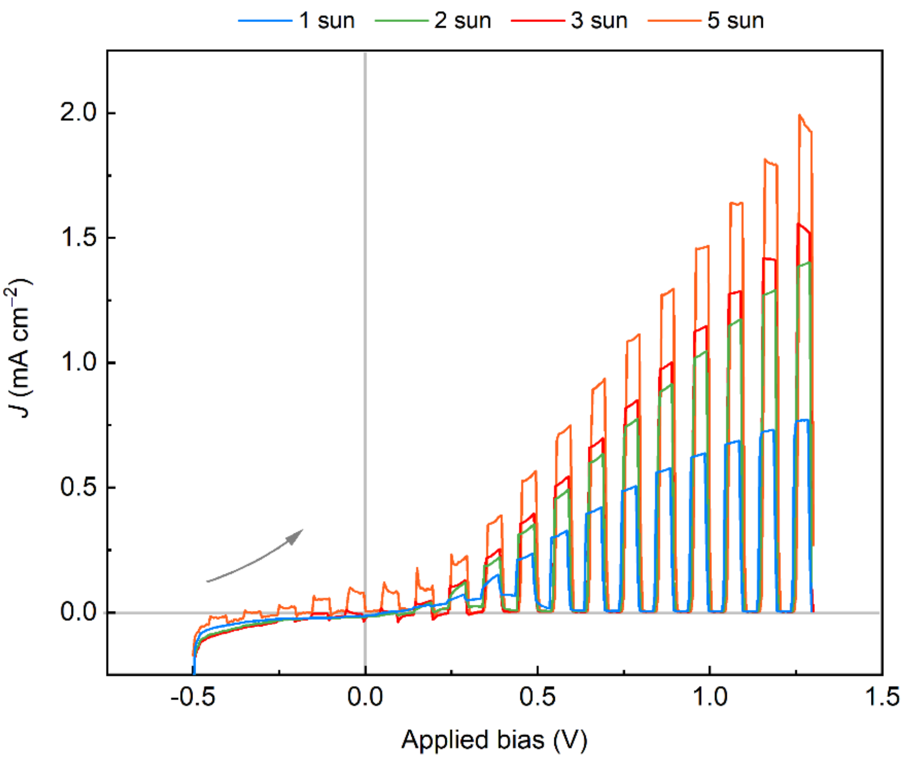

Figure S10. Linear sweep voltammetry (LSV) traces of Pt-$BiVO_4$ under chopped light irradiation. Experiments were performed in a two-electrode setup, in 0.1 M KB_i, 0.1 M K_2SO_4 buffer solution (pH 8.5, temperature between 34 – 61 °C). The temperature of the water bath was maintained at 25 °C.

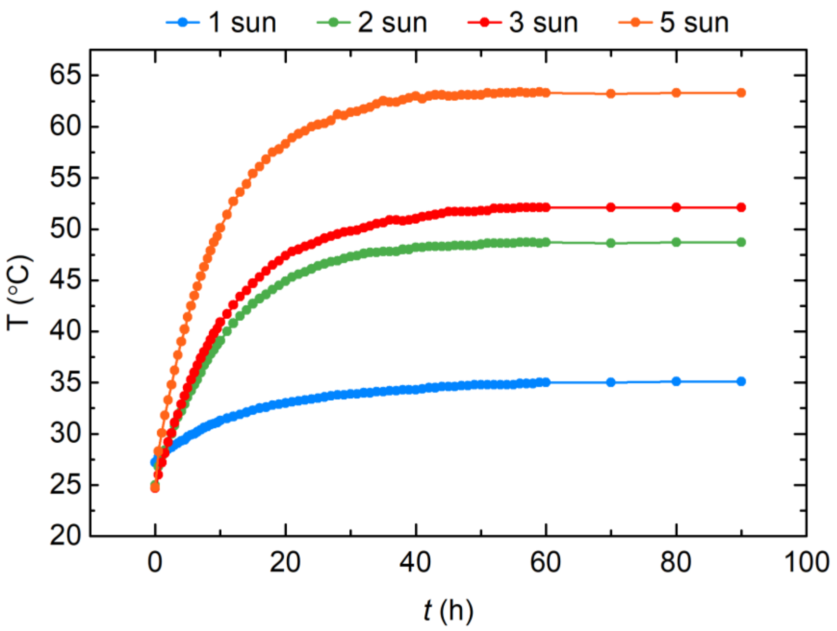

Figure S11. Temperature of the electrolyte solution during operation of a Pt-TE-PVK-$BiVO_4$ setup. The 3D-printed water bath was equipped with an additional cooling block to maintain a steady water temperature under concentrated irradiation.

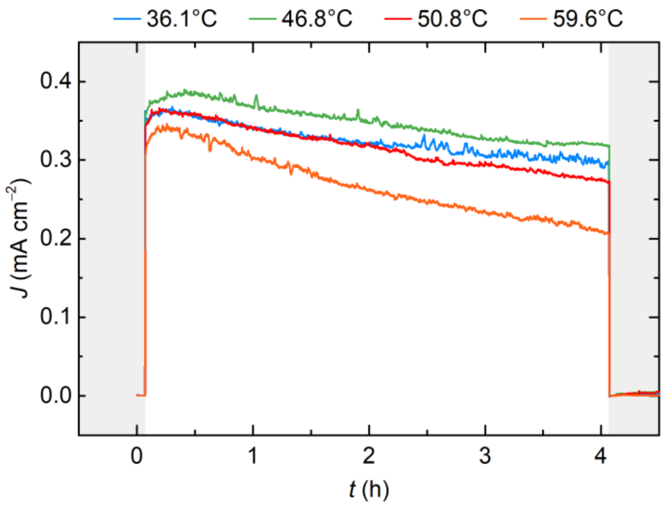

Figure S12. Water splitting experiments with Pt-$BiVO_4$ under different electrolyte temperatures. All 4 h CPE experiments were conducted under 1 sun irradiation and 1.0 V applied bias (0.1 M KB_i, 0.1 M K_2SO_4, pH 8.5). The electrolyte temperature was set to 36.1-59.6 °C by an external temperature-controlled water bath, to simulate the solution heating achieved under 1-5 sun irradiation. These control experiments show no significant changes in the water splitting photocurrents at elevated electrolyte temperatures.

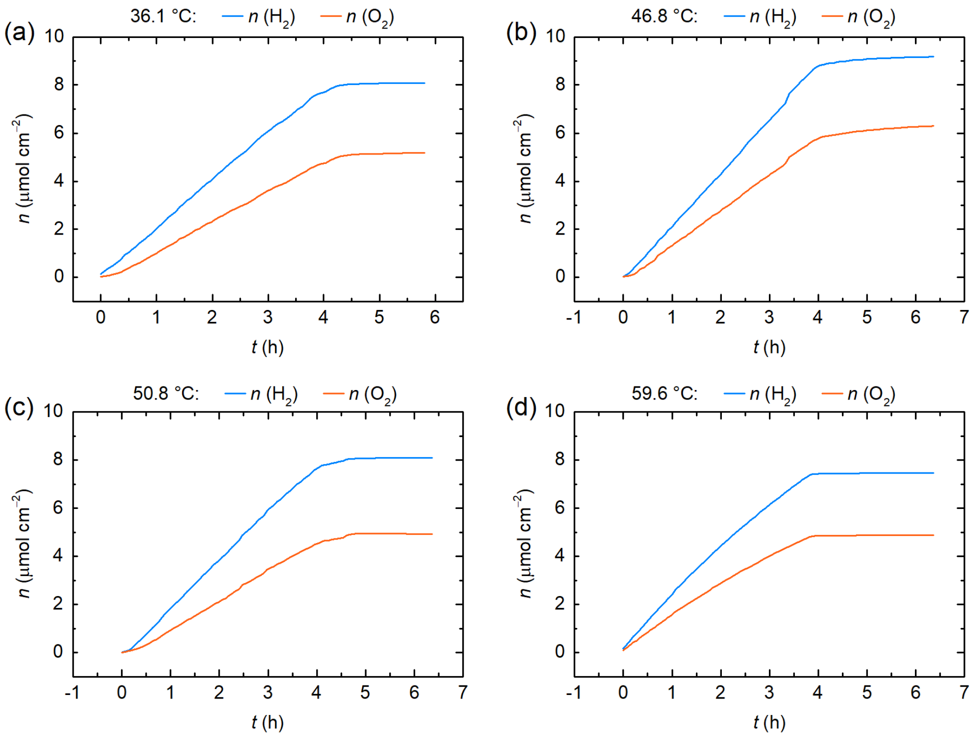

Figure S13. Product evolution from Pt-$BiVO_4$ under different electrolyte temperatures. (a-d) H_2 and O_2 quantification of 4 h CPE experiments under given temperature (1 sun, 1.0 V applied bias, 0.1 M KB_i, 0.1 M K_2SO_4, pH 8.5). H_2 and O_2 quantification was performed in flow by automated gas sampling from the reactor headspace into a gas chromatograph. These control experiments show no variation in the amount of products with the electrolyte temperature.

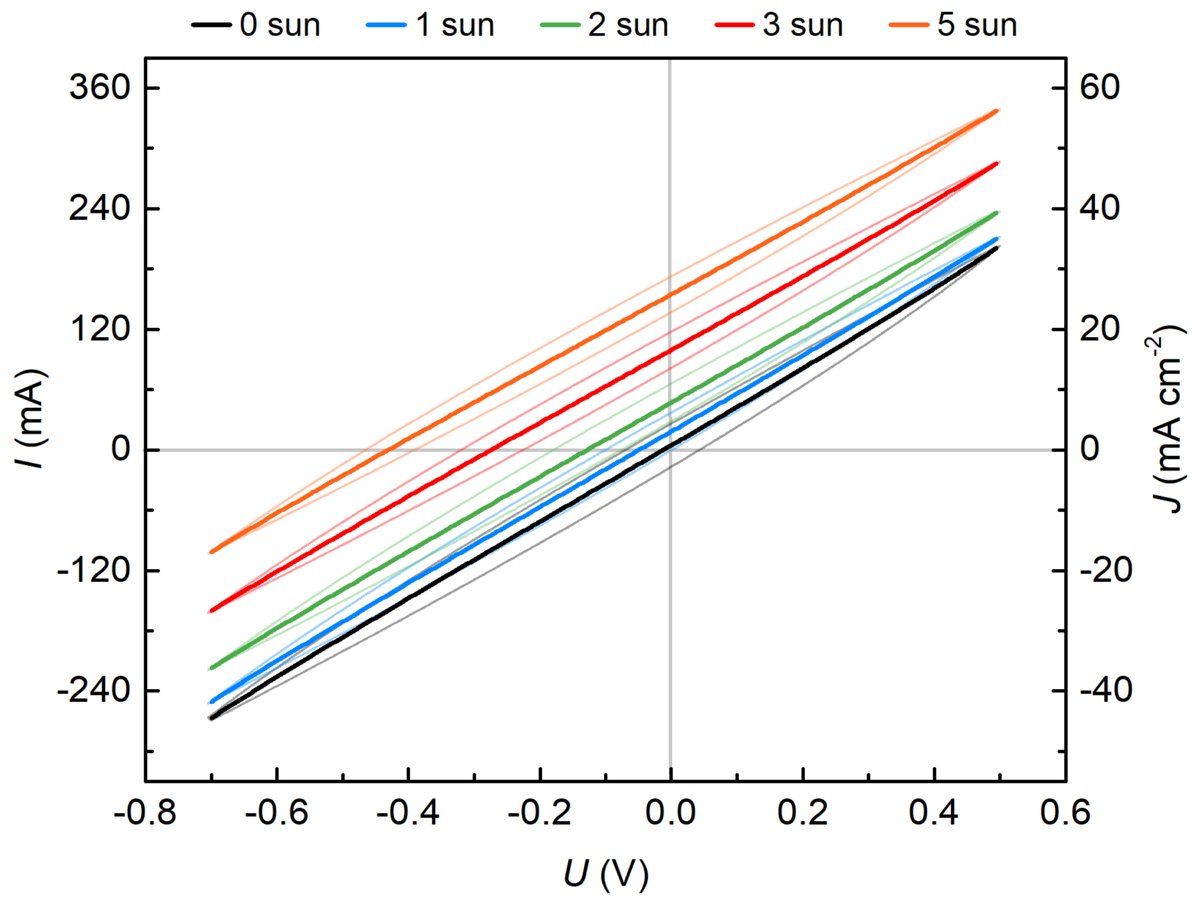

Figure S14. *I-V* curve of a TEG under 1-5 sun irradiation. The faint lines reveal the hysteresis between forward and backward scans, which is due to the rapid temperature change under applied voltage (Peltier effect). The solid traces represent the average between forward and backward scans, and follow the expected linear behaviour. *I-V* curves were recorded by connecting the potentiostat directly to the TEG in a Pt-TE-$BiVO_4$ assembly (34 – 61 °C solution temperature; 25 °C water bath temperature), at a 100 mV s^{-1} scan rate. The current density was calculated based on the 6 cm^{-2} photoactive areas of $BiVO_4$ and PVK. Data reveals the photocurrent density of neither Pt-TE-$BiVO_4$ nor Pt-TE-PVK-$BiVO_4$ is limited by the TEG output.

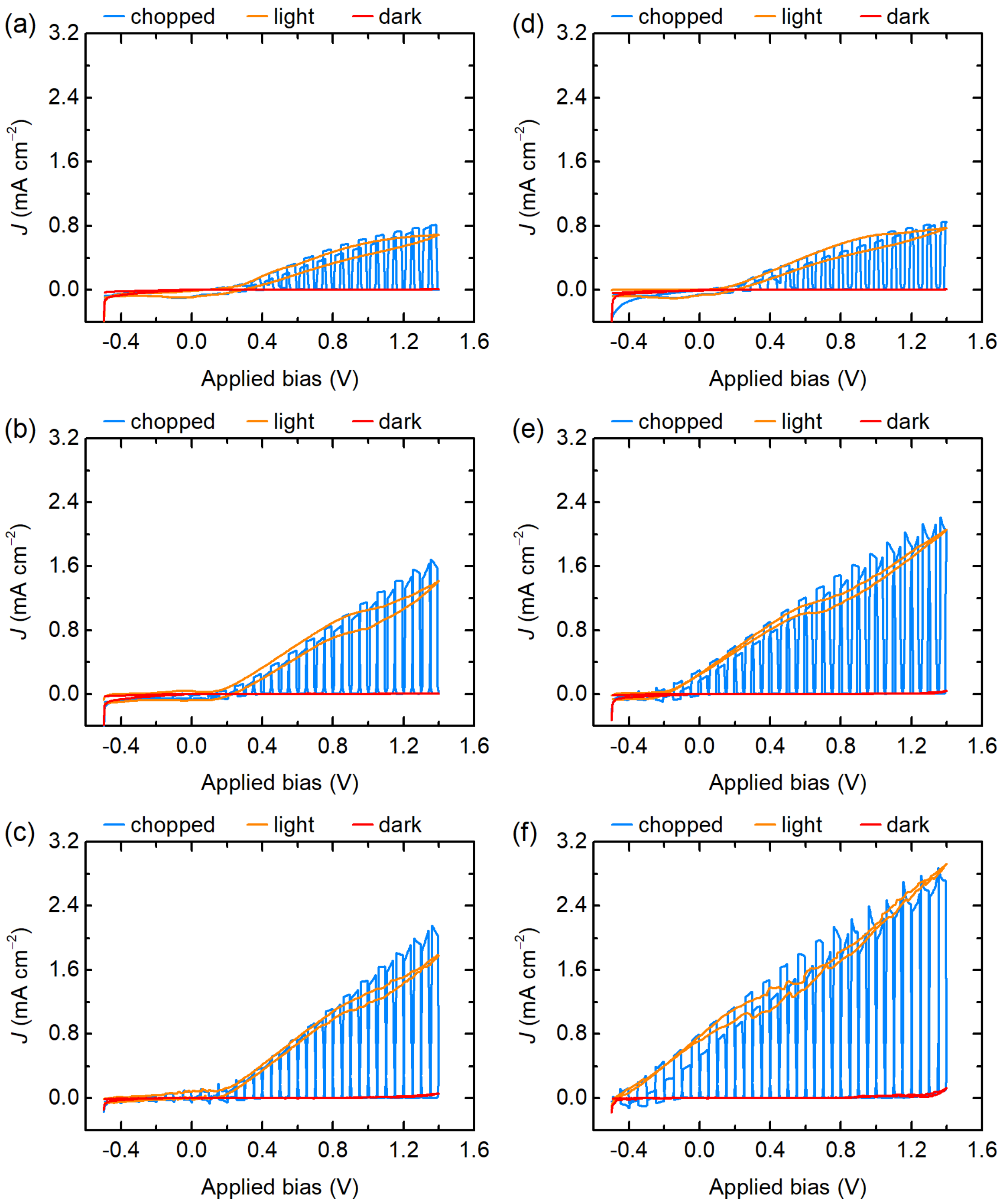

Figure S15. Cyclic voltammetry (CV) traces of the Pt-TE-$BiVO_4$ system under varying light concentration. (a-c) Pt-$BiVO_4$ assembly. (d-f) Pt-TE-$BiVO_4$ setup. Traces are recorded under chopped, continuous and no light irradiation: (a, d) - 1 sun, (b, e) - 3 sun, (c, f) - 5 sun.

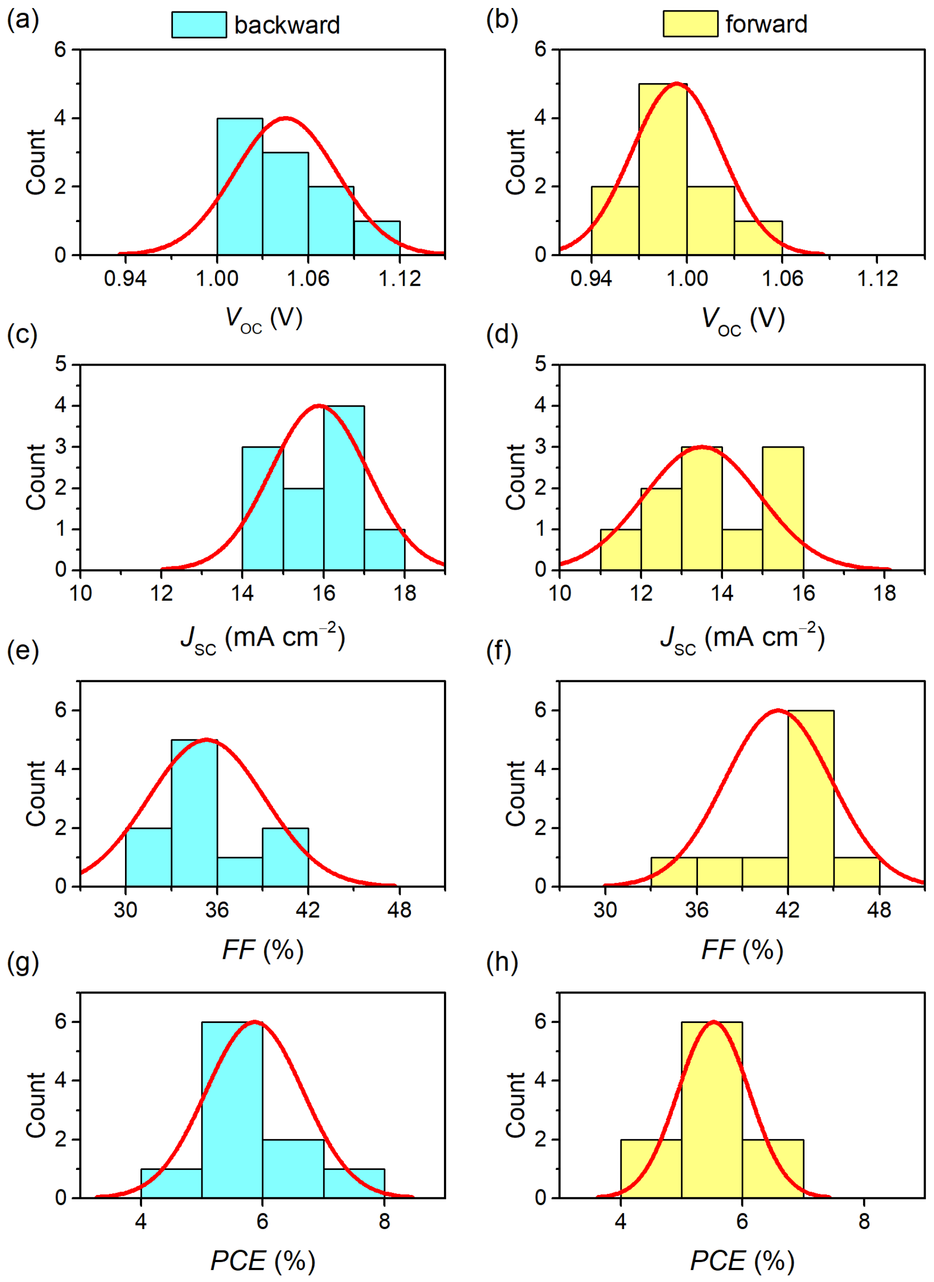

Figure S16. Histograms revealing the photovoltaic performance of the 6 cm^2 inverse-structure perovskite devices fabricated in this study. (a, b) Open circuit voltage (V_{OC}). (c, d) Short circuit current density (J_{SC}). (e, f) Fill factor (FF). (g, h) Photovoltaic cell efficiency (PCE) ((a, c, e, g) - backward, (b, d, f, h) - forward scan direction; normal distribution curves are depicted in red). The 10 devices display average values of 1.05±0.03 V V_{OC}, –15.9±1.2 mA cm^{-2} J_{SC}, 35.3±3.8% FF, 5.9±0.8% PCE in backward scan direction, and 0.99±0.03 V V_{OC}, –13.5±1.4 mA cm^{-2} J_{SC}, 41.4±3.5% FF, 5.5±0.6% PCE for the forward scans.

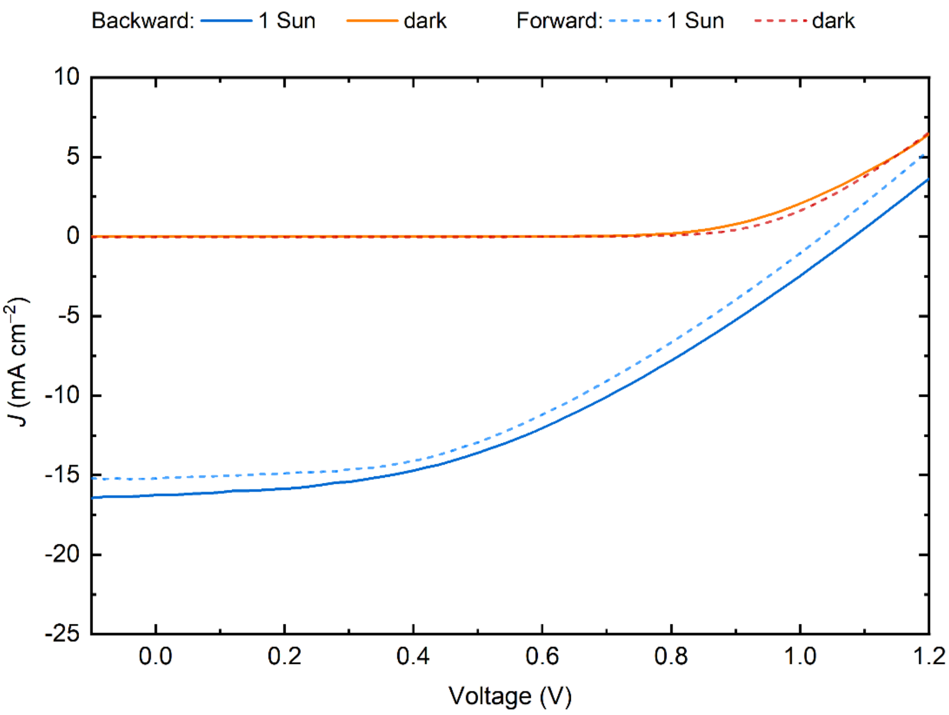

Figure S17. *J-V* traces of the champion 6 cm^2 perovskite device. The device displays a 1.083 V V_{OC}, −16.3 mA cm^{-2} J_{SC}, 41.1% FF and 7.24% PCE in backward scan direction, and 1.034 V V_{OC}, −15.2 mA cm^{-2} J_{SC}, 42.7% FF and 6.73% PCE in forward scan direction (1 sun, AM1.5G, 100 mW cm^{-2}).

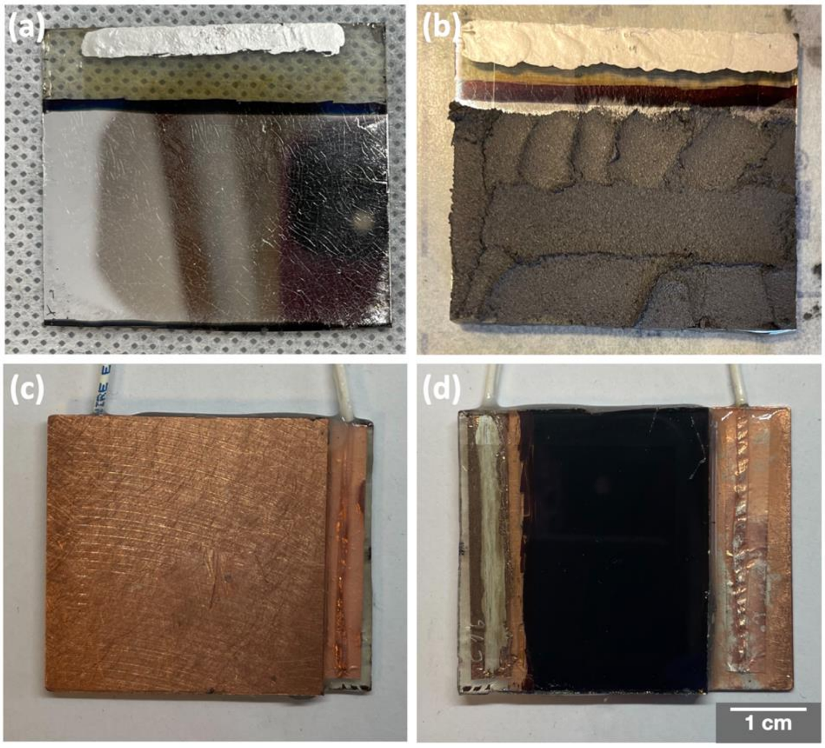

Figure S18. PVK encapsulation procedures. (a) A photograph of as-prepared inverse-structure perovskite cells. (b) Graphite epoxy (GE) paste application step where the GE was spread on top of the Ag contact. (c), (d) A photograph of fully encapsulated PVK.

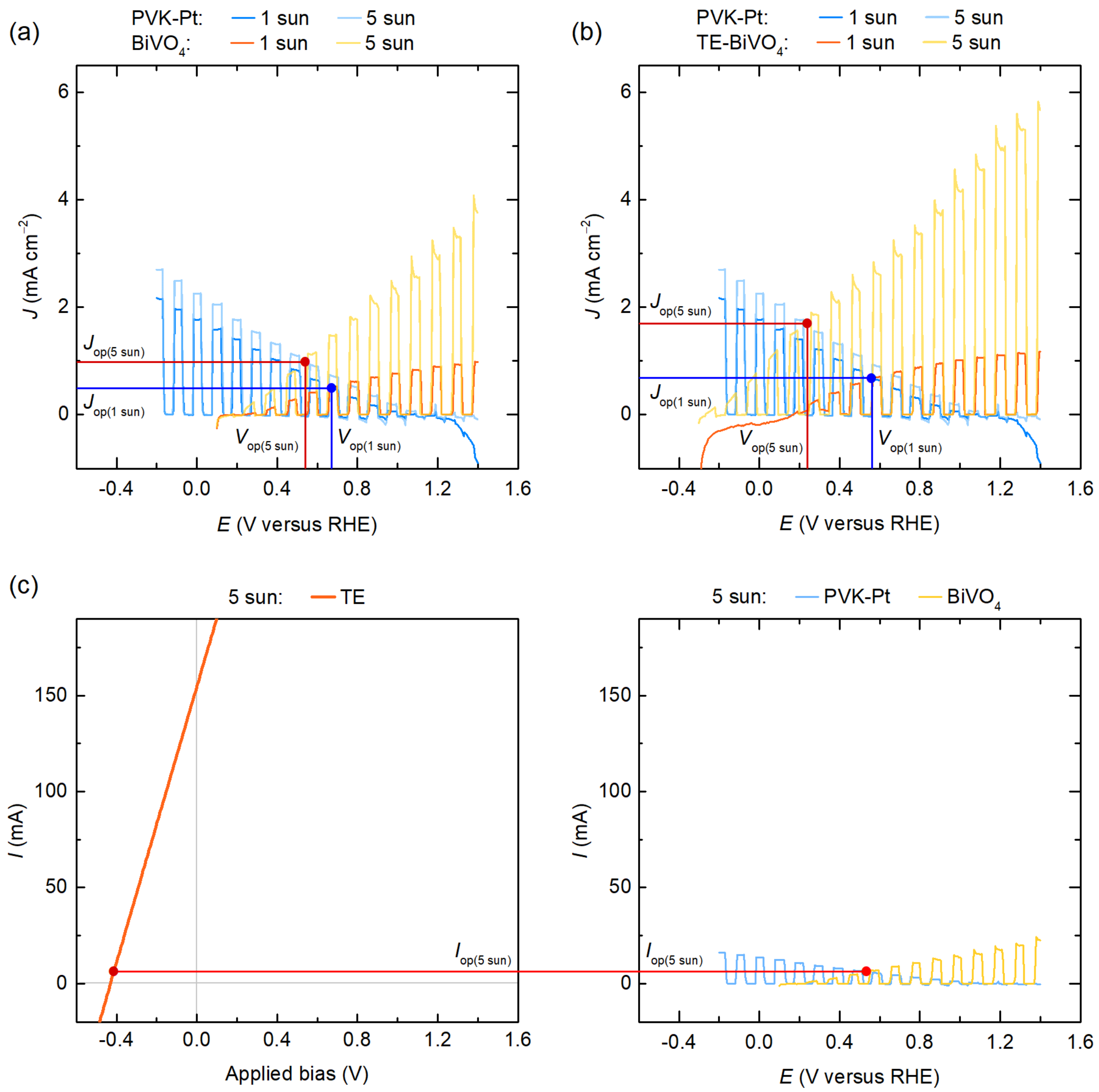

Figure S19. Overlap between the LSV traces of PVK-Pt, TE-$BiVO_4$ and $BiVO_4$. (a) Intersection between the LSV curves of PVK-Pt and $BiVO_4$ under 1 and 5 sun irradiation. (b) LSV traces of PVK-Pt and TEG-$BiVO_4$ under 1 and 5 sun irradiation. In this configuration, the perovskite PV cell is wired to a Pt cathode, as encountered in case of a buried PV photocathode.[6] The sign of the PVK-Pt electrode signal is reversed, to illustrate the photocurrent overlap. LSV scans were measured in a three-electrode setup with a Ag/AgCl reference and Pt counter electrode, under chopped light irradiation (0.1 M KB_i, 0.1 M K_2SO_4, pH 8.5). (c) Current matching between TE, PVK-Pt and $BiVO_4$ components under 5 sun irradiation. *I*-*V* and LSV traces are plotted on the same numerical scales, to illustrate that both light absorbers are the current limiting components. The PVK-Pt and $BiVO_4$ traces overlap at an absolute photocurrent of around 6 mA, which is close to the open circuit potential of the TE element. As a result, the TE element can supply an additional photovoltage above 0.4 V, whereas its electrical resistance of only 2.6 Ω does not affect the overall photocurrent. The resistance is calculated as the slope of the averaged *I*-*V* trace under no irradiation from Fig. 2a, to account for heating hysteresis.

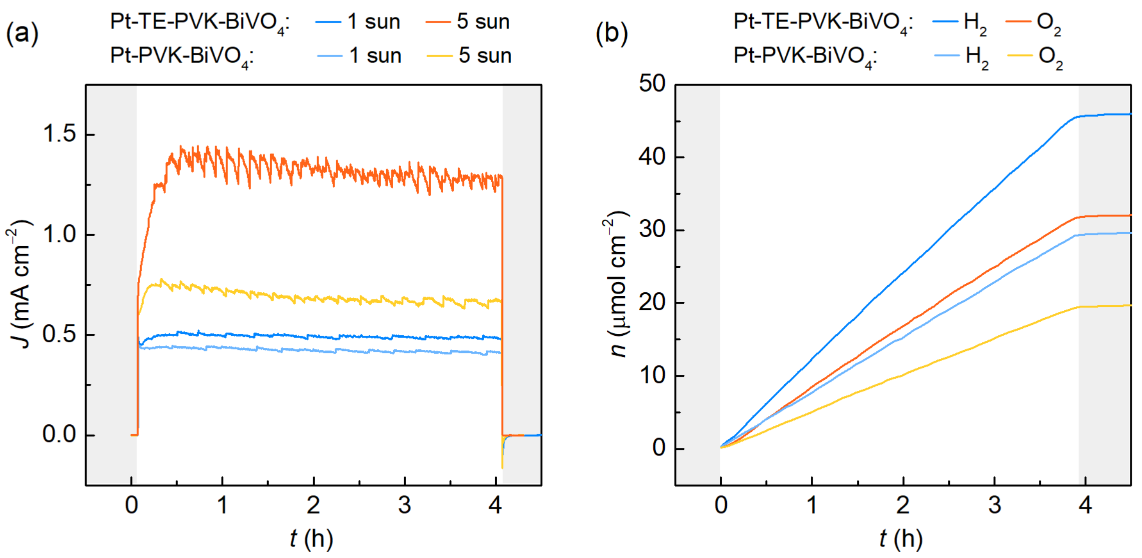

Figure S20. Performance of the Pt-TE-PVK-$BiVO_4$ system under concentrated light irradiation. (a) Representative CPE traces of bias-free water splitting under 1 and 5 sun irradiation. (b) Product evolution during 4 h CPE, under 5 sun irradiation.

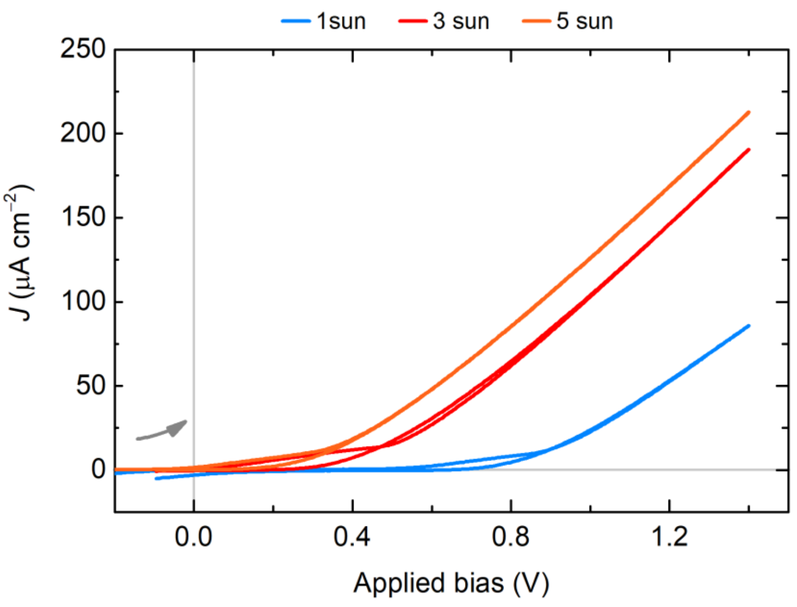

Figure S21. CV scans of an integrated Pt-TE-Fe_2O_3 system for water splitting under concentrated irradiation. The grey arrow indicates the scan start and direction. The shift in the onset bias with increased light intensity corresponds to the one observed for the Pt-TE-$BiVO_4$ system. However, data indicates that overall unassisted water splitting cannot be achieved in case of the Pt-TE-Fe_2O_3 arrangement.

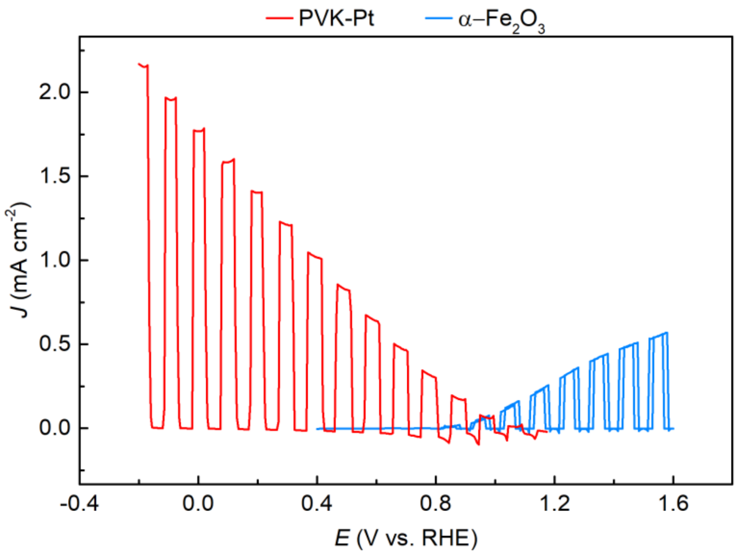

Figure S22. LSV traces of 6 cm^2 PVK-Pt and 1 cm^2 Fe_2O_3 electrodes. The PVK-Pt signal is reversed to illustrate the photocurrent overlap. Traces are recorded in a three-electrode configuration, in 1 M NaOH.

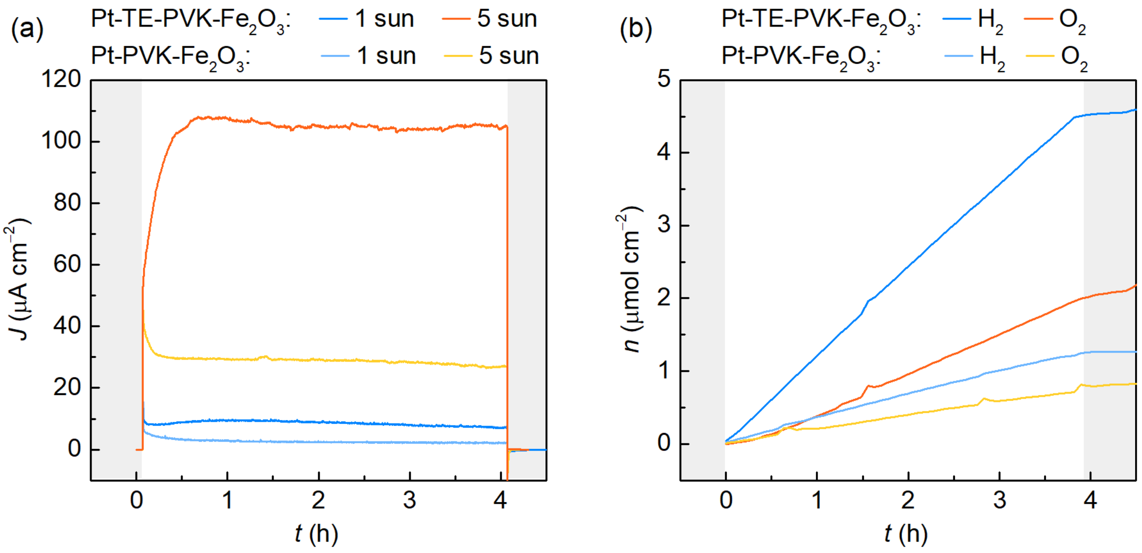

Figure S23. Behaviour of the Pt-TE-PVK-Fe_2O_3 system under concentrated light irradiation. (a) 4 h CPE traces of unassisted water splitting under 1 and 5 sun irradiation. (b) Product evolution under 5 sun irradiation.

Table S1. Electrolyte, water bath temperature and Seebeck voltage in the PEC reactor at different solar concentrations. The average steady-state values are recorded after 1 h irradiation for temperature, and between 1-2 h irradiation for the Seebeck voltage. Chronopotentiometry was performed at $I = 0$ mA in a two-electrode setup.

Light intensity (sun)	$T_{electrolyte}$ (°C)	$T_{water\ bath}$ (°C)	ΔT (°C)	Seebeck voltage (mV)
1	36.1 ± 1.1	26.9 ± 0.1	9.2 ± 1.1	78.4 ± 0.3
2	48.6 ± 1.8	27.3 ± 0.1	21.3 ± 1.8	287.5 ± 1.1
3	50.8 ± 1.0	27.2 ± 0.1	23.6 ± 1.0	371.2 ± 1.3
5	59.6 ± 3.3	27.4 ± 0.2	32.2 ± 3.3	457.2 ± 0.9

Table S2. Recent reports on bias-free solar water splitting devices. Abbreviations: PC – photocatalyst, PEC – photoelectrocatalyst, PV – photovoltaic, PVK – perovskite, KB_i – potassium borate buffer, KP_i – potassium phosphate buffer.

System	Type	STH (%)	Irradiation (sun)	Stability (h)	Area (cm^2)	Electrolyte solution	Ref.
**Single light absorber**							
$Rh/Cr_2O_3/CoOOH-SrTiO_3:Al$	PC suspension	0.65	1	12.5	n.a.	distilled H_2O	[9]
$Rh/Cr_2O_3/MnO_x$ -tetragonal zircon $BiVO_4$	PC suspension	0.012	1	12	n.a.	distilled H_2O	[10]
$Cr_2O_3/Rh/IrO_2-Y_2Ti_2O_5S_2$	PC suspension	0.007	1	20	n.a.	distilled H_2O buffered by La_2O_3 (pH 8.5)	[11]
Pt – TE – $BiVO_4$|TiCo	PEC	0.08	3	≥4	6.0	0.1 M KB_i, K_2SO_4 (pH 8.5)	this work
Pt – TE – $BiVO_4$|TiCo	PEC	0.11	5	≥ 4	6.0	0.1 M KB_i, K_2SO_4 (pH 8.5)	this work
**Dual light absorbers**							
PVK|GE|Pt – $BiVO_4$|TiCo	tandem PEC	1.3	1	10	0.25	0.1 M KB_i, K_2SO_4 (pH 8.5)	[6]
Pt – TE – PVK – $BiVO_4$|TiCo	tandem PV-PEC	0.59	1	≥ 4	6	0.1 M KB_i, K_2SO_4 (pH 8.5)	this work
Pt – TE – PVK – $BiVO_4$|TiCo	tandem PV-PEC	0.31	5	≥ 4	6	0.1 M KB_i, K_2SO_4 (pH 8.5)	this work
Pt|TiO_2|SiHJ – PE-$BiVO_4$|FeOOH|NiOOH	tandem PEC	3.5	1	10	4	KB_i(pH 9)/ HClO4(pH0)	[12]
Pt|TiO_2|CdS|Sb_2Se_3 – Mo:$BiVO_4$|NiFeO	tandem PEC	1.5	1	10	0.36	0.5 M KP_i + 0.01 M V_2O_5 (pH 7.0)	[13]
Pt|$CuBi_2O_4$ – Mo:$BiVO_4$|Co-Pi	tandem PEC	0.15	1	≤ 1	0.32	0.1 M KP_i (pH 7.0)	[14]
Ru-loaded $SrTiO_3$:La,Rh/carbon/$BiVO_4$:Mo	Z-scheme PC panel	1.0	1	17	7.5	distilled H_2O	[15]

Table S3. Photoelectrochemical performance of the unassisted Pt-TE-$BiVO_4$ system for overall solar water splitting. Product amounts (*n*) are given after 4 h CPE at no applied bias. STH is calculated assuming 100% FY.

Light intensity (sun)	$J_{steady\text{-}state}$ (mA cm^{-2})	$n(H_2)$ (µmol cm^{-2})	$n(O_2)$ (µmol cm^{-2})	STH (%)
1	0.001 ± 0.000	0.02 ± 0.01	–	–
2	0.10 ± 0.005	2.72 ± 0.86	2.14 ± 0.58	0.06 ± 0.01
3	0.20 ± 0.021	5.09 ± 2.01	3.52 ± 1.39	0.08 ± 0.03
5	0.45 ± 0.028	12.16 ± 3.94	7.72 ± 2.24	0.11 ± 0.03

Table S4. PEC performance of the Pt-TE-PVK-$BiVO_4$ assembly for unassisted water splitting. STH is calculated assuming 100% FY.

Light intensity (sun)	$J_{steady\text{-}state}$ (mA cm^{-2})	$n(H_2)$ (µmol cm^{-2})	$n(O_2)$ (µmol cm^{-2})	STH (%)
1	0.48 ± 0.02	22.60 ± 0.51	15.90 ± 0.81	0.59 ± 0.02
3	1.00 ± 0.06	39.95 ± 1.38	29.94 ± 1.03	0.41 ± 0.08
5	1.27 ± 0.10	47.53 ± 3.84	36.29 ± 3.64	0.31 ± 0.13

Table S5. PEC performance of Pt-PVK-$BiVO_4$ tandem devices for overall water splitting (STH assuming 100% FY).

Light intensity (sun)	$J_{steady\text{-}state}$ (mA cm^{-2})	$n(H_2)$ (µmol cm^{-2})	$n(O_2)$ (µmol cm^{-2})	STH (%)
1	0.42 ± 0.02	19.91 ± 0.72	13.52 ± 0.22	0.51 ± 0.03
3	0.66 ± 0.04	29.23 ± 1.49	20.16 ± 1.77	0.27 ± 0.05
5	0.73 ± 0.08	30.52 ± 1.87	21.72 ± 1.84	0.18 ± 0.09

Table S6. PEC performance of the unassisted Pt-TE-PVK-Fe_2O_3 system for overall water splitting (STH assuming 100% FY).

Light intensity (sun)	$J_{steady\text{-}state}$ (μA cm^{-2})	$n(H_2)$ (μmol cm^{-2})	$n(O_2)$ (μmol cm^{-2})	STH (%)
1	9.08 ± 0.54	0.43 ± 0.04	0.67 ± 0.28	0.011 ± 0.001
3	77.32 ± 7.79	3.72 ± 0.72	1.65 ± 0.70	0.032 ± 0.010
5	105.36 ± 0.67	4.99 ± 0.50	2.30 ± 0.11	0.026 ± 0.001

Table S7. Photoelectrochemical data of Pt-PVK-Fe_2O_3 unassisted devices for overall solar water splitting (STH assuming 100% FY). O_2 amounts were partly below the detection threshold under 1 and 3 sun irradiation.

Light intensity (sun)	$J_{steady\text{-}state}$ (μA cm^{-2})	$n(H_2)$ (μmol cm^{-2})	$n(O_2)$ (μmol cm^{-2})	STH (%)
1	3.55± 1.64	0.18 ± 0.09	0.27	0.004 ± 0.002
3	24.18± 3.32	1.06 ± 0.09	0.34	0.010 ± 0.004
5	28.47± 0.71	1.36 ± 0.12	0.74 ± 0.13	0.007 ± 0.001

Supplementary References

1. Lai, Y. H.; Palm, D. W.; Reisner, E., Multifunctional coatings from scalable single source precursor chemistry in tandem photoelectrochemical water splitting. *Adv. Energy Mater.* **2015,** *5,* 1501668.

2. Kim, T. W.; Choi, K.-S., Nanoporous $BiVO_4$ photoanodes with dual-layer oxygen evolution catalysts for solar water splitting. *Science* **2014,** *343*, 990-994.

3. Lai, Y. H.; Lin, C.-Y.; Lv, Y.; King, T. C.; Steiner, A.; Muresan, N. M.; Gan, L.; Wright, D. S.; Reisner, E., Facile assembly of an efficient CoO_x water oxidation electrocatalyst from Co-containing polyoxotitanate nanocages. *Chem. Commun.* **2013,** *49*, 4331-4333.

4. Xiao, J.; Huang, H.; Huang, Q.; Li, X.; Hou, X.; Zhao, L.; Ma, R.; Chen, H.; Li, Y., Remarkable improvement of the turn–on characteristics of a Fe_2O_3 photoanode for photoelectrochemical water splitting with coating a FeCoW oxy–hydroxide gel. *Appl. Catal. B* **2017,** *212*, 89-96.

5. Andrei, V.; Hoye, R. L. Z.; Crespo-Quesada, M.; Bajada, M.; Ahmad, S.; De Volder, M.; Friend, R.; Reisner, E., Scalable triple cation mixed halide perovskite–$BiVO_4$ tandems for bias-free water splitting. *Adv. Energy Mater.* **2018,** *8*, 1801403.

6. Pornrungroj, C.; Andrei, V.; Rahaman, M.; Uswachoke, C.; Joyce, H. J.; Wright, D. S.; Reisner, E., Bifunctional perovskite-$BiVO_4$ tandem devices for uninterrupted solar and electrocatalytic water splitting cycles. *Adv. Funct. Mater.* **2021**, 2008182.

7. Sahm, C. D.; Ucoski, G. M.; Roy, S.; Reisner, E., Automated and continuous-flow platform to analyze semiconductor–metal complex hybrid systems for photocatalytic CO_2 reduction. *ACS Catal.* **2021,** *11*, 11266-11277.

8. Kudo, A.; Omori, K.; Kato, H., A novel aqueous process for preparation of crystal form-controlled and highly crystalline $BiVO_4$ powder from layered vanadates at room temperature and its photocatalytic and photophysical properties. *J. Am. Chem. Soc.* **1999,** *121*, 11459-11467.

9. Takata, T.; Jiang, J.; Sakata, Y.; Nakabayashi, M.; Shibata, N.; Nandal, V.; Seki, K.; Hisatomi, T.; Domen, K., Photocatalytic water splitting with a quantum efficiency of almost unity. *Nature* **2020,** *581*, 411-414.

10. Dai, D.; Liang, X.; Zhang, B.; Wang, Y.; Wu, Q.; Bao, X.; Wang, Z.; Zheng, Z.; Cheng, H.; Dai, Y., Strain adjustment realizes the photocatalytic overall water splitting on tetragonal zircon $BiVO_4$. *Adv. Sci.* **2022**, 2105299.

11. Wang, Q.; Nakabayashi, M.; Hisatomi, T.; Sun, S.; Akiyama, S.; Wang, Z.; Pan, Z.; Xiao, X.; Watanabe, T.; Yamada, T., Oxysulfide photocatalyst for visible-light-driven overall water splitting. *Nat. Mater.* **2019,** *18*, 827.

12. Feng, S.; Wang, T.; Liu, B.; Hu, C.; Li, L.; Zhao, Z. J.; Gong, J., Enriched surface oxygen vacancies of photoanodes by photoetching with enhanced charge separation. *Angew. Chem. Int. Ed.* **2020,** *59*, 2044-2048.

13. Yang, W.; Kim, J. H.; Hutter, O. S.; Phillips, L. J.; Tan, J.; Park, J.; Lee, H.; Major, J. D.; Lee, J. S.; Moon, J., Benchmark performance of low-cost Sb_2Se_3 photocathodes for unassisted solar overall water splitting. *Nat. Commun.* **2020,** *11*, 861.

14. Kim, J. H.; Adishev, A.; Kim, J.; Kim, Y. S.; Cho, S.; Lee, J. S., All-bismuth-based oxide tandem cell for solar overall water splitting. *ACS Appl. Energy Mater.* **2018,** *1*, 6694-6699.

15. Wang, Q.; Hisatomi, T.; Suzuki, Y.; Pan, Z.; Seo, J.; Katayama, M.; Minegishi, T.; Nishiyama, H.; Takata, T.; Seki, K.; Kudo, A.; Yamada, T.; Domen, K., Particulate photocatalyst sheets based on carbon conductor layer for efficient Z-scheme pure-water splitting at ambient pressure. *J. Am. Chem. Soc.* **2017,** *139*, 1675-1683.
